# Exploring the Efficacy of Four Apiaceae Essential Oils against Nine Stored-Product Pests in Wheat Protection

**DOI:** 10.3390/plants13040533

**Published:** 2024-02-15

**Authors:** Nickolas G. Kavallieratos, Nikoleta Eleftheriadou, Maria C. Boukouvala, Anna Skourti, Constantin S. Filintas, Demeter Lorentha S. Gidari, Filippo Maggi, Paolo Rossi, Ettore Drenaggi, Mohammad Reza Morshedloo, Marta Ferrati, Eleonora Spinozzi

**Affiliations:** 1 Laboratory of Agricultural Zoology and Entomology, Department of Crop Science, Agricultural University of Athens, 75 Iera Odos Str., 11855 Athens, Greece; nikolelef@aua.gr (N.E.); mbouk@aua.gr (M.C.B.); annaskourti@aua.gr (A.S.); p1172219@aua.gr (C.S.F.); dimitralorentha@gmail.com (D.L.S.G.); 2 Chemistry Interdisciplinary Project (ChIP), School of Pharmacy, University of Camerino, Via Maddona Delle Carceri, 62032 Camerino, Italy; filippo.maggi@unicam.it (F.M.); marta.ferrati@unicam.it (M.F.); eleonora.spinozzi@unicam.it (E.S.); 3 School of Bioscience and Veterinary Medicine, University of Camerino, Via Gentile III Da Varano, 62032 Camerino, Italy; paolo.rossi@unicam.it; 4 Department of Horticultural Science, Faculty of Agriculture, University of Maragheh, Maragheh 5518183111, Iran; morshedlooreza@gmail.com

**Keywords:** botanical insecticides, green pesticides, stored-product insects, biological activities

## Abstract

The Apiaceae family, known for aromatic plants producing bioactive essential oils (EOs), holds significance across sectors, including agrochemicals. This study evaluated the insecticidal potential of four Apiaceae EOs from *Crithmum maritimum* L., *Trachyspermum ammi* (L.) Sprague ex Turrill, *Smyrnium olusatrum* L., and *Elwendia persica* (Boiss.) Pimenov and Kljuykov against various significant storage pests (*Sitophilus oryzae* (L.), *Trogoderma granarium* Everts, *Rhyzopertha dominica* (F.), *Tribolium castaneum* (Herbst), *T. confusum* Jacquelin du Val, *Oryzaephilus surinamensis* (L.), *Alphitobius diaperinus* (Panzer), *Acarus siro* L., and *Tenebrio molitor* L.) on wheat. Insect mortality rates were monitored at intervals of 1, 2, 3, 4, 5, 6, and 7 days. *Smyrnium olusatrum* EO exhibited the highest efficacy, followed by *T. ammi*, *C. maritimum*, and *E. persica* EOs, although efficacy varied by species, developmental stage, and concentration. Notably, complete mortality occurred for several pests at 1000 ppm of *S. olusatrum* and *T. ammi* EOs. Gas chromatography–mass spectrometry (GC–MS) analysis revealed key compounds in these EOs, including myrcene, germacrone, and curzerene in *S. olusatrum* EO, and thymol, γ-terpinene, and p-cymene in *T. ammi* EO. These findings emphasize their potential as botanical insecticides. *Smyrnium olusatrum* and *T. ammi* EOs emerge as promising eco-friendly pest management options due to their efficacy, highlighted compound composition, and availability of biomass from both wild and cultivated sources.

## 1. Introduction

The substantial dependence on synthetic pesticides for managing arthropod pests, particularly within food supplies, presents notable environmental as well as human health hazards [1,2]. These hazards encompass mammalian toxicity, environmental pollution, and bioaccumulation. Furthermore, the frequent emergence of resistance among insect species to prevalent compounds significantly constrains their efficacy [3]. Amidst this concern, the European Union advocates for a considerable decrease in chemical pesticide usage, integrating innovative concepts and advanced technologies aligned with Integrated Pest Management (IPM) tenets [4,5].

*Rhyzopertha dominica* (F.), *Sitophilus oryzae* (L.), *Tribolium castaneum* (Herbst), *Trogoderma granarium* Everts, *Alphitobius diaperinus* (Panzer), *Tribolium confusum* Jacquelin du Val, *Oryzaephilus surinamensis* (L.), *Acarus siro* L., and *Tenebrio molitor* L. are all noxious arthropods of stored products [6,7,8,9,10,11,12]. These pests inhabit locations associated with agriculture, such as farms, storage facilities, grain bins, mills, warehouses, and grain elevators. They also dwell in bakeries, food shops, pet stores, and retail establishments [8]. They are accountable for both qualitative and quantitative deterioration of affected commodities, as well as for instigating severe allergic reactions in humans and animals [13,14,15,16,17,18,19,20,21]. In the past years, various chemical insecticides have been utilized to manage the aforementioned pests. However, accomplishing this task proves to be challenging due to their heightened tolerance, posing difficulties in effective control [22,23,24,25,26,27,28,29,30].

In recent years, multiple investigations have been carried out to assess the efficacy of natural pesticidal formulations, such as diatomaceous earth, entomopathogenic nematodes and fungi, nanoemulsions (NEs), microemulsions, and essential oils (EOs), as grain protectants against different pests of storages, as well as other pests, exhibiting promising results [31,32,33,34,35,36,37,38,39]. Extracted from various plant parts like leaves, seeds, roots, and flowers, EOs represent secondary plant metabolites with substantial applications in aromatherapy, perfumery, and cosmetics industry [40]. These EOs and their constituents hold valuable potential in diverse fields such as agriculture, environment, and human health. Their significance extends further as they have attracted considerable attention for the development of insecticides and acaricides [41,42,43]. Indeed, research efforts have extensively concentrated on assessing the contact effects of EOs on insect species by application onto filter paper, or by topical administration onto the dorsal region of the insect thorax, while also investigating their efficacy as botanical repellents [44,45,46,47,48]. Various EOs and their physiologically active constituents have garnered significant recommendations as promising contact toxicants and fumigants because of their volatile characteristics, leaving negligible residual impact [49]. Among several botanical sources of EOs, the family of Apiaceae is one of the most important worldwide for the high economic value and cultivation systems developed in temperate and Mediterranean regions [50]. Apiaceae EOs find applications in food and beverages (alcoholic and non-alcoholic drinks), pharmaceutics, cosmetics, and perfumery. Recently, the agrochemical industry has been investigating these products as potential candidates within the scenario of replacing synthetic pesticides with safer and eco-friendly alternatives (e.g., the farm-to-fork strategy of the EU).

*Crithmum maritimum* L., found extensively along Western Europe’s Mediterranean coastlines, is a facultative halophyte renowned for its diverse culinary and practical applications [51]. The leaves boast significant antioxidant and nutritional properties, while the seeds contain valuable edible oil and secondary compounds with promising industrial uses [52]. A biennial plant, *Smyrnium olusatrum* L., found across the Mediterranean region from North Africa to the British Isles, is distinguished by the plentiful presence of sesquiterpenes equipped with a furan ring [53]. Among these compounds, isofuranodiene predominates. This plant potentially exhibits neuritogenic, anti-inflammatory, and anti-cancer effects, along with insecticidal and acaricidal properties [54]. The Persian *Trachyspermum ammi* (L.) Sprague ex Turrill, referred to as ajwain, is cultivated for its fruits. This species contains around 2–4.4% brown EO rich in thymol, widely used in treating gastrointestinal issues, bronchial problems, and lack of appetite. This EO demonstrates anti-aggregatory, antimicrobial, and fungicidal effects. Ajwain serves as traditional remedy for various human and animal ailments, possessing stimulant, antispasmodic, and carminative properties [55,56,57]. *Elwendia persica* (Boiss.) Pimenov and Kljuykov (syn. *Bunium persicum* (Boiss.) B.Fedtsch.), found in dry temperate regions of Central Asia like India, Kazakhstan, Afghanistan, Pakistan, Egypt, and Iran, yields fruits serving as spice, antiseptic, and carminative agents. Studies revealed that its EO primarily comprises γ-terpinene, cuminaldehyde, and *p*-cymene [58,59]. Additionally, major antioxidants such as *p*-coumaric acid, caffeic acid, and kaempferol are isolated from the fruit’s polar fraction [60]. The plant nomenclature follows POWO [61].

The insecticidal efficacy of EOs derived from *C. maritimum*, *S. olusatrum*, *T. ammi*, and *E. persica* has been previously assessed against various pests of the storage environment using different application methods such as fumigation, NEs, and direct aqueous solution application [62,63,64,65,66,67,68,69]. Despite the potential displayed by these EOs, their efficacy is often limited to a single developmental stage of the targeted pest, such as adults or larvae, and never as grain protectants. Hence, there remains a pressing need for research into novel insecticidal formulations capable of eradicating multiple developmental stages of either one or multiple storage pests when applied directly to grains. Thus, the objective of the current investigation was to assess the pesticidal impact of EOs derived from *C. maritimum*, *S. olusatrum*, *T. ammi*, and *E. persica* against different developmental stages of nine storage pests known for their detrimental impact, including the understudied species *A. diaperinus*, *A. siro*, *T. granarium*, and *T. molitor*, over multiple time intervals.

## 2. Results

### 2.1. EO Chemical Compositions

The chemical compositions of *C. maritimum*, *T. ammi*, *E. persica*, and *S. olusatrum* EOs were determined through GC–MS analyses. The results revealed monoterpene hydrocarbons as the predominant chemical class in all cases, accounting for 75.0, 60.7, 49.2, and 53.6% of the total composition for *T. ammi*, *C. marithimum*, *S. olusatrum*, and *E. persica* EOs, respectively. Oxygenated monoterpenes were also found in high amounts in the EOs obtained from Iranian species. On the contrary, *S. olusatrum* EO was particularly rich in furanosesquiterpenes, representing 27.0% of the total detected compounds. In this case, oxygenated monoterpenes were found in lower percentages (21.2%), followed by oxygenated sesquiterpenes (2.7%). Thymol (38.9%) stood out as the predominant oxygenated monoterpene within *T. ammi* EO, surpassing the abundance of the other twelve identified compounds. Moreover, *γ*-terpinene and *p*-cymene constituted a great part of the EO, achieving 28.8 and 28.0% of the total composition, respectively. Regarding *E. persica*, the total chemical composition has been described by Perinelli et al. [70]. Briefly, *γ*-terpinene (35.8%) represented the major constituent. The oxygenated monoterpenes cumin aldehyde (16.6%), *α*-terpinen-7-al (14.0%), and *γ*-terpinen-7-al (11.7%) were also found in high amounts. The EO of *C. marithimum* was mainly composed of *γ*-terpinene (36.1%). Other compounds detected at noteworthy levels were *β*-phellandrene (14.7%), dillapiole (12.0%), thymol methyl ether (11.2%), and *p*-cymene (10.1%). The predominant monoterpene hydrocarbons of *S. olusatrum* EO were myrcene (29.4%) and *β*-phellandrene (15.3%). Germacrone (20.5%), which belongs to the chemical class of oxygenated sesquiterpenes, was also detected in high percentages. Moreover, among furanosesquiterpenes, the analysis revealed the presence of curzerene (16.3%), isofuranodiene (6.2%), and furanoeremophil-1-one (4.0%) (Table 1).

### 2.2. Effectiveness against Alphitobius Diaperinus Larvae and Adults

Between and within exposures, all main effects and respective interactions were significant for both adults and larvae of *A. diaperinus* (Table 2). After one day of exposure, *S. olusatrum* resulted in 28.9 and 45.6% mortality of *A. diaperinus* larvae at 500 and 1000 ppm, respectively, while pirimiphos-methyl only reached 8.9% mortality. At 500 ppm, *S. olusatrum* caused more than 68% mortality after seven days of exposure, while at 1000 ppm, complete mortality (100%) was observed after six days of exposure, significantly outperforming pirimiphos-methyl at both concentrations. Following *S. olusatrum*, *C. maritimum* EO caused 13.3 and 44.4% mortality to *A. diaperinus* larvae, while the remaining EOs had no effect on mortality. Concerning *A. diaperinus* adults, there was no effect on mortality observed for any EO at 500 ppm, while at 1000 ppm, *C. maritimum* EO caused 13.3% mortality, followed by *T. ammi*, exhibiting 6.7% mortality after 7 days of exposure (Table 3).

### 2.3. Effectiveness against Tribolium Castaneum Larvae and Adults

For *T. castaneum*, between and within exposures, all main effects and the respective interactions were significant for both adults and larvae (Table 2). At 500 ppm, *S. olusatrum*, *T. ammi*, and *E. persica* EOs caused *T. castaneum* larvae 17.8, 10.0, and 3.3% mortality after one day of exposure, respectively, while *C. maritimum* had no effect on mortality. After seven days of exposure, however, the aforementioned EOs caused 77.8, 61.1, 48.9, and 67.8% mortality, respectively, with mortality caused by *S. olusatrum* (77.8%) significantly higher than that of pirimiphos–methyl (56.7%). After four days of exposure, *S. olusatrum* EO resulted in complete mortality of *T. castaneum* larvae, followed by *T. ammi* (93.3%), *E. persica* (65.7%), and *C. maritimum* (61.1%), all significantly outperforming pirimiphos–methyl (34.4%). Complete mortality was observed for *T. ammi* EO after five days of exposure, while at the last exposure interval, *C. maritimum* caused 91.1% mortality, followed by *E. persica* at 90.0% mortality. Adults of *T. castaneum* were only affected by *S. olusatrum* EO three days post-exposure at 500 ppm. Mortality reached 13.3% at the final exposure interval, while pirimiphos–methyl failed to affect mortality at all exposure intervals. At 1000 ppm, *S. olusatrum* EO caused *T. castaneum* adults 26.7% mortality after two days of exposure, reaching 81.1% mortality after seven days, followed by *E. persica* (13.3%), *T. ammi* (5.6%), and *C. maritimum* (5.6%) (Table 4).

### 2.4. Effectiveness against Tribolium Confusum Larvae and Adults

All main effects were significant between exposures for both *T. confusum* larvae and adults, whereas their respective combination was significant only for larvae. For both larvae and adults, within exposures, all main effects and respective interactions were significant (Table 2). At 500 ppm, after two days of exposure, all EOs affected the mortality of *T. confusum* larvae, with *S. olusatrum* resulting in the highest mortality (51.1%), followed by *T. ammi* (41.1%), *E. persica* (15.6%), and *C. maritimum* (3.3%). After seven days of exposure, *T. ammi* EO (80.0%) outperformed *S. olusatrum* (65.6%), followed by *E. persica* (50.0%) and *C. maritimum* (36.7%). After four days of exposure, *T. ammi* EO resulted in complete mortality of *T. confusum* larvae, followed by *S. olusatrum* (86.7%), *E. persica* (60.0%), and *C. maritimum* (48.9%), with the first two EOs exhibiting significantly higher mortalities than pirimiphos–methyl (43.3%). After seven days of exposure, *T. ammi* EO remained the most effective, showcasing complete mortality, followed by *S. olusatrum* (96.7%), *E. persica* (94.4%), and *C. maritimum* (82.2%), with pirimiphos–methyl exhibiting significantly lower mortalities than the first three EOs (68.9%). Regarding *T. confusum* adults, pirimiphos–methyl had no effect on mortality at any exposure interval, while only *E. persica* EO had a low effect on mortality ranging from 2.2 to 4.4% after three and seven days of exposure, respectively, at 500 ppm. Very low mortality was recorded only for *C. maritimum* EO (2.2%) after one day of exposure at 1000 ppm. *Trachyspermum ammi* EO had no effect on mortality after five days of exposure, while after six days of exposure exhibited lower mortality (2.2%) compared to *S. olusatrum* (8.9%), *E. persica* (7.8%), and *C. maritimum* (5.6%). Nevertheless, at seven days post-exposure, *T. ammi* EO caused the highest mortality rate (13.3%), followed by *S. olusatrum* (10.0%), *E. persica* (8.9%), and *C. maritimum* (7.8%) (Table 5).

### 2.5. Effectiveness against Tenebrio Molitor Larvae and Adults

For *T. molitor*, between exposures, all main effects and respective interactions were significant for both larvae and adults. Within exposures, exposure × concentration was not significant for larvae and adults, and exposure × concentration × EO type was not significant for larvae (Table 2). Regarding *T. molitor* larvae, only *S. olusatrum* EO affected mortality at 500 ppm, which ranged from 3.3 to 11.1% from four to seven days, respectively. At 1000 ppm, *S. olusatrum* EO caused 2.2–18.9% larval mortality from two to seven days of exposure, followed by *C. maritimum* reaching 4.4% mortality after seven days, and *E. persica* (2.2%), while *T. ammi* did not affect mortality at any exposure interval. Adults of *T. molitor* were mostly affected by *T. ammi* EO, which resulted in 13.3–100% mortality from one to six days of exposure at 500 ppm, significantly outperforming pirimiphos–methyl (7.8–51.1%). Following *T. ammi* EO, *S. olusatrum* and *E. persica* exhibited 75.6 and 7.8% mortality after seven days of exposure, respectively, while *C. maritimum* did not affect the mortality of *T. molitor* adults at all exposure intervals. At 1000 ppm, *T. ammi* EO remained the most efficient, exhibiting 27.8–100% after one to four days, respectively, significantly outperforming pirimiphos–methyl at every exposure interval (10.0–35.6%, respectively). *Smyrnium olusatrum* EO caused 96.7% mortality after seven days of exposure, followed by *E. persica* (26.7%), while *C. maritimum* only caused 1.1% mortality (Table 6).

### 2.6. Effectiveness against Trogoderma Granarium Larvae and Adults

All main effects and respective interactions were significant for both adults and larvae between exposures. Within exposures, only exposure × concentration was not significant for larvae of *T. granarium* (Table 2). Larvae of *T. granarium* were only affected by *S. olusatrum* and *T. ammi*, exhibiting 3.3% mortality for both EOs after one day of exposure at 500 ppm, which reached 50.0 and 41.1% for each EO, respectively, after seven days of exposure, significantly higher than pirimiphos–methyl (26.7%). The remaining EOs did not affect mortality at any exposure interval. At 1000 ppm, *S. olusatrum* EO exhibited the highest efficacy against larvae of *T. granarium*, exhibiting 13.3 to 93.3% mortality after one to seven days of exposure, remaining significantly higher than pirimiphos–methyl (0.0–25.6%, respectively). Following this EO, *T. ammi* exhibited 68.9% mortality after seven days of exposure, while *C. maritimum* and *E. persica* only reached 17.8 and 4.4%, respectively. Concerning the adults of *T. granarium*, mortalities between one day (18.9%) and two days (40.0%) post-exposure were significantly higher when treated with *T. ammi* at 500 ppm, reaching 78.9% at seven days post-exposure. A significant increase in mortalities was also observed for *S. olusatrum* EO, exhibiting 27.8–70.0% mortalities after two to four days after exposure, respectively, ultimately reaching 81.1% mortality after seven days of exposure, outperforming *T. ammi.* The remaining EOs, *C. maritimum*, and *E. persica*, reached 48.9 and 42.2% mortality at the last exposure interval, respectively. At 1000 ppm, complete mortality was achieved by *S. olusatrum* EO after four days of exposure, followed by *T. ammi* (88.9%), both significantly higher than pirimiphos–methyl (42.2%). After seven days of exposure, all EOs exhibited significantly higher mortality rates than pirimiphos–methyl (70.0%), with *T. ammi* resulting in complete mortality, followed by *C. maritimum* (91.1%) and *E. persica* (87.8%) (Table 7).

### 2.7. Effectiveness against Oryzaephilus Surinamensis Larvae and Adults

For *O. surinamensis*, all main effects and respective interactions were significant for both adults and larvae between exposures. Within exposures, only exposure × concentration and exposure × concentration × EO type were not significant for adults (Table 2). Larvae of *O. surinamensis* exhibited the highest susceptibility to *S. olusatrum*, resulting in mortalities ranging from 23.3 to 57.8% after one to seven days of exposure at 500 ppm. Following *S. olusatrum* EO, *T. ammi* caused up to 53.3% mortality after seven days of exposure, with *C. maritimum* (26.7%) and *E. persica* (6.7%) following. Mortalities caused by *S. olusatrum* and *T. ammi* EOs were significantly higher than pirimiphos–methyl after two days of exposure, exhibiting 72.2 and 47.8%, respectively, compared to the latter (8.9%) at 1000 ppm. Complete mortality was achieved by *S. olusatrum* EO after five days of exposure, while *T. ammi* resulted in 100% mortality after six days of exposure. At the final exposure interval, *C. maritimum* and *E. persica* EOs resulted in 61.1% and 53.3% mortalities, respectively. *O. surinamensis* adults suffered higher mortalities to *S. olusatrum* (21.1%), *T. ammi* (12.2%), and *C. maritimum* (10.0%) than *E. persica* EO (8.9%) after two days at 500 ppm. All EOs exhibited significantly higher mortalities than pirimiphos–methyl from three to seven days of exposure at 500 ppm. Mortalities reached 48.9, 43.3, 41.1, and 33.3% for *T. ammi*, *C. maritimum*, *S. olusatrum*, and *E. persica* EOs, respectively, at seven days post-exposure. A similar trend was observed between the EOs at 1000 ppm. *Elwendia persica* EO caused low (10.0%) mortality after one day of exposure, while *T. ammi* did not affect mortality. However, after two days of exposure, *S. olusatrum*, *T. ammi*, and *C. maritimum* EOs outperformed *E. persica*, exhibiting 36.7, 26.7, and 25.6% mortalities, respectively, compared to the later (22.2%). All, nevertheless, demonstrated significantly higher mortalities than pirimiphos–methyl (2.2%) at the same exposure interval. At the final exposure interval, *C. maritimum* EO resulted in the highest mortality (77.8%), followed by *T. ammi* (76.7%), *S. olusatrum* (75.6%), and *E. persica* (65.6%), although the only significant difference among treatments was observed between all EOs and pirimiphos–methyl (11.1%) (Table 8).

### 2.8. Effectiveness against Rhyzopertha Dominica Adults

Between exposures, all main effects and respective interactions were significant for *R. dominica* adults. Within exposures, only exposure × concentration was not significant (Table 2). At 500 ppm, *S. olusatrum* was the most effective EO against *R. dominica* adults, exhibiting 41.1% mortality after seven days of exposure, followed by *E. persica* (24.4%), while *T. ammi* did not affect mortality at any exposure interval. At 1000 ppm, after one day of exposure, *S. olusatrum* EO exhibited the highest mortality (24.4%), followed by *C. maritimum* (18.9%) and *T. ammi* (17.8%), all significantly outperforming pirimiphos–methyl (4.4%), while *E. persica* demonstrated 6.7% mortality. After seven days of exposure, *S. olusatrum* EO remained the most efficient, causing 95.6% mortality. *Crithmum maritimum*, *E. persica*, and *T. ammi* EOs followed with 68.9, 56.7, and 45.6% mortalities, respectively (Table 9).

### 2.9. Effectiveness against Sitophilus Oryzae Adults

For *S. oryzae* adults, between exposures, all main effects and respective interactions were significant. Within exposures, only exposure × concentration was not significant (Table 2). At 500 ppm, *S. oryzae* adults exhibited 20.0 and 3.3% mortality when treated with *S. olusatrum* and *C. maritimum*, respectively, after one day of exposure, while the remaining EOs did not affect mortality. At seven days post-exposure, *S. olusatrum* and *C. maritimum* EOs caused 90.0 and 33.3% mortalities, respectively, followed by *E. persica* (6.7%). No effect on mortality was observed for *T. ammi* EO at any exposure interval at 500 ppm. At 1000 ppm, *C. maritimum* caused 94.4% after the last exposure interval. *Smyrnium olusatrum* EO resulted in complete mortality after three days of exposure, significantly higher than pirimiphos–methyl (38.9%). *Elwendia persica* EO affected mortality at four days of exposure (3.3%), ultimately reaching 11.1% mortality at the last exposure interval. Mortality caused by *T. ammi* remained low from one to seven days of exposure, exhibiting 3.3 to 8.9% mortality, respectively (Table 10).

### 2.10. Effectiveness against Acarus Siro Nymphs and Adults

Between exposures, all main effects were significant for *A. siro* nymphs and adults, apart from concentration in the case of adults. The respective interaction was insignificant. Within exposures, exposure × concentration and exposure × concentration × EO type were not significant for nymphs and adults (Table 2). At 500 ppm, *A. siro* nymphs were affected only by *S. olusatrum* and *T. ammi* EOs after one day of exposure, exhibiting low mortalities (1.1% for both EOs). After seven days of exposure, *S. olusatrum* EO resulted in the highest mortality rates, demonstrating 55.6%, followed by *T. ammi* (47.8%), *C. maritimum* (17,8%), and *E. persica* (6.7%), with mortalities gradually increasing over time. At 1000 ppm, a similar trend was observed after seven days of exposure, with *S. olusatrum* EO remaining the most efficient (63.3%), followed by *T. ammi* (56.7%), *C. maritimum* (32.2%), and *E. persica* (15.6%). Regarding *A. siro* adults, all *S. olusatrum*, *T. ammi*, and *C. maritimum* EOs exhibited 2.2% mortality at 500 ppm after one day of exposure, while *E. persica* did not affect mortality. At the final exposure interval, *S. olusatrum* EO resulted in the highest mortality rate (67.8%), followed by *T. ammi* (55.6%), *C. maritimum* (26.7%), and *E. persica* (15.6%). At 1000 ppm, *S. olusatrum*, *T. ammi*, and *C. maritimum* EOs exhibited 4.4, 3.3, and 3.3% mortalities, respectively, after one day of exposure, whereas *E. persica* did not affect mortality. After seven days of exposure, the aforementioned EOs caused 76.7, 65.6, and 45.6%, respectively, with *E. persica* remaining less effective, causing 23.3% mortality (Table 11).

## 3. Discussion

Generally, EO chemical composition is influenced by diverse factors, such as climate, altitude, growth conditions, soil type, agricultural practices, and harvesting time [71]. The chemical composition identified in the *E. persica* EO aligns with previously reported compositions of EOs derived from wild Iranian plants. These typically showcase a prevalence of γ-terpinene, γ-terpinen-7-al, and cumin aldehyde [72,73,74,75]. On the contrary, the identified chemical composition differs from the one reported for cultivated Iranian *E. persica*, in which cumin aldehyde, *α*-terpinen-7-al, and *γ*-terpinen-7-al levels are usually higher than those of *γ*-terpinene [76]. Regarding *S. olusatrum* EO, its chemical composition differs from that reported by Quassinti et al. [77]. A lower percentage of isofuranodiene was detected, together with a higher one of the monoterpenes myrcene and *β*-phellandrene. Isofuranodiene is a thermosensitive compound and undergoes Cope rearrangement under conventional gas chromatographic runs, producing curzerene [78]. For this reason, the values derived by GC–MS are usually underestimated. Moreover, the chemical composition of the EO could be influenced by the geographic origin of the plant used in this study, which differs from the one used by Quassinti et al. [77]. Regarding *C. maritimum* EO, its chemical composition aligned with that reported by Piatti et al. [79] concerning major EO constituents. However, our study highlighted a lower percentage of γ-terpinene and a higher content of dillapiole. The chemical composition of *C. maritimum* EO is influenced by diverse factors, and many chemotypes have already been reported [80]. On the contrary, *T. ammi* EO was mainly dominated by thymol, *γ*-terpinene, and *p*-cymene, and this result is in line with the chemical compositions already reported in the literature [81].

Amidst botanical pesticides, EOs have garnered escalating interest due to their notable effectiveness coupled with their characteristic of low persistence in the environment [82,83,84]. Moreover, EOs often present low toxicity to non-target organisms and mammals [35,85,86]. In addition, these plant-derived products are frequently distinguished by possessing diverse modes of action, thereby diminishing the likelihood of resistance development, a noteworthy characteristic in the context of IPM [86,87]. In the current research, multiple EOs have demonstrated their insecticidal potential against diverse pests exhibiting tolerance to conventional insecticides. Among all EOs, *S. olusatrum* was the most efficient, followed by *T. ammi*, *C. maritimum*, and *E. persica*, although the effectiveness of each EO varied depending on the species, developmental stage, and concentration applied. Past studies indicate that *C. maritimum* exhibited insecticidal effects against *S. oryzae*, *T. confusum*, *T. castaneum*, *R. dominica*, and *O. surinamensis* when applied on filter paper, or for the evaluation of fumigant and contact toxicity [65,66,67]. The effectiveness of *S. olusatrum-*derived isofuranodiene and *S. olusatrum* NE against *T. granarium*, *T. molitor*, *T. confusum*, and *T. castaneum* has been previously investigated [68,88]. *Trachyspermum ammi* EOs have been tested against *A. siro*, *T. granarium*, *T. molitor*, *T. confusum*, *T. castaneum*, *O. surinamensis*, *R. dominica*, and *S. oryzae* as fumigants, repellants, or NEs on wheat [63,64,69], except for *A. diaperinus*. As for *E. persica* EO, its impact has only been tested for its repellent effect and contact toxicity against *T. castaneum* [62].

In general, most EOs tested here had little to no effect against *A. diaperinus* adults; nevertheless, *S. olusatrum* EO had a remarkable effect on larvae, causing complete mortality after six days of exposure at 1000 ppm, significantly outperforming pirimiphos–methyl. However, EOs tested here had little to no effect against adults of this pest. Interestingly, the mortality rates of *A. diaperinus* larvae after five and seven days of exposure to *S. olusatrum* EO at 1000 ppm were identical to the resulting mortality rates exhibited by Kavallieratos et al. [89] when treating larvae with the concentration of deltamethrin indicated on the formulation label. The difference in mortality rates between larvae and adults may be partially attributed to differences in structural and physiological characteristics of the cuticle across different insect life stages [90,91].

A noteworthy discovery of the current investigation is the efficiency of several tested EOs in controlling *Tribolium* spp., since both *T. confusum* and *T. castaneum* have been recognized for decades as highly tolerant species to numerous insecticides [34,92,93,94]. Both *S. olusatrum* and *T. ammi* EO demonstrated complete mortality of *T. castaneum* larvae after five and four days, respectively, at 1000 ppm, with *S. olusatrum* EO also exhibiting high mortality rates for adults, whereas pirimiphos–methyl could not control the species in this developmental stage. The elevated mortality in adults of *T. castaneum* holds profound significance since adults are more tolerant than larvae [95,96,97]. Regarding *T. confusum*, larval mortality was high for all EOs tested at both concentrations, with *T. ammi* EO resulting in complete larval mortality after four days of exposure at the high concentrations applied. In line with *T. castaneum*, *T. confusum* adults also exhibit higher tolerance compared to larvae [95]. This has become evident in the present investigation, since the EOs applied had little effect on adult mortality, whereas no effect was observed for pirimiphos–methyl. Nevertheless, previous investigations on the effectiveness of EO derived from *Carlina acaulis* L. against *T. confusum* exhibited remarkable results after two days of exposure in both larvae and adults of this species [98], demonstrating that the various modes of action of different EOs are reflected in the observed mortality rates.

In general, EOs demonstrate insufficient efficacy in controlling *T. molitor* larvae. For example, the application of *Tanacetum vulgare* L. EO on wheat caused 8.9% mortality of *T. molitor* larvae at seven days post-exposure [99]. The low effectiveness of EOs against larvae of this pest has become evident in the current investigation as well. Nevertheless, remarkable adult mortality rates were observed in *T. molitor* adults when treated with *T. ammi* and *S. olusatrum* EOs. Complete mortality was induced by the former at both concentrations, while the latter achieved a 96.7% mortality rate at 1000 ppm. These specific EOs have exhibited exceptional efficacy against *T. molitor* adults compared to other botanical formulations. For instance, after seven days of initial exposure, the following EOs resulted in varying mortality rates: *Santalum album* L. EO exhibited a rate of 60.0%, *Melaleuca cajuputi* Powell EO showed 50.0%, *Syzygium aromaticum* (L.) Merr. and L. M. Perry EO demonstrated 43.3%, *Copaifera officinalis* L. EO revealed 46.7%, *Corymbia citriodora* (Hook.) K. D. Hill and L. A. S. Johnson EO displayed 40.0%, *Thymus vulgaris* L. EO showcased 40.0%, *Boswellia carteri* Birdw. EO presented 33.3%, *Coriandrum sativum* L. EO showed 30.0%, *Elettaria cardamomum* (L.) Maton EO exhibited 23.3%, *Pogostemon cablin* (Blanco) Benth. EO illustrated 21.1%, and *T. vulgare* EO presented 52.2% [69,99,100].

Various insecticidal formulations based on β-cyfluthrin, deltamethrin, or the bacteriocin microcin extracted from *Citrobacter* spp. have highlighted a higher tolerance among larvae of *T. granarium* compared to adults [27,101,102]. In the present study, *S. olusatrum* and *T. ammi* EOs exhibited remarkable efficacy for the management of both larvae and adults of this species, with *S. olusatrum* EO demonstrating exceptionally high to complete suppression of both developmental stages at 1000 ppm, significantly outperforming pirimiphos–methyl. Hence, these EOs might serve as viable alternatives to synthetic organophosphorus insecticides such as pirimiphos–methyl.

In the current study, all EOs exhibited exceptional efficiency against *O. surinamensis*, with *S. olusatrum* and *T. ammi* EOs successfully suppressing larvae of this species, and all EOs demonstrated high mortality rates for adults (65.6–77.8%), in both cases significantly outperforming pirimiphos–methyl. Alternative insecticides yielded notably lower efficacy. For instance, *T. vulgare* EO resulted in the mortality of 93.3% of larvae and 13.3% of adults [99]. On the other hand, *Mentha longifolia* (L.) Huds. EO-based NE caused the death of 86.7% of larvae and 63.3% of adults of *O. surinamensis* at 1000 ppm, following a seven-day exposure to treated wheat [103]. When subjected to single and combined treatments with two concentrations of the entomopathogenic nematode *Steinernema carpocapsae* (Weiser) (50 IJs/cm^2^, 100 IJs/cm^2^) and the entomopathogenic fungus *Beauveria bassiana* (Bals. -Criv.) Vuill. (1 × 10^6^ conidia/mL), mortalities of *O. surinamensis* adults ranged from 9.38 to 48.64% at the same exposure interval [38].

Concerning the effectiveness of the tested EOs against adults of *R. dominica*, all treatments resulted in elevated mortalities at 1000 ppm and seven days post-exposure, ranging from 45.6 to 95.6%. These are rather elevated mortality rates considering that *S. carpocapsae* (50 IJs/cm^2^, 100 IJs/cm^2^) and *B. bassiana* (1 × 10^6^ conidia/mL) alone and combined resulted in 25.43–71.22% mortality of this pest after seven days of exposure [38]. Nevertheless, *C. acaulis* EO at 1000 ppm resulted in complete mortality of *R. dominica* adults at four days post-exposure [98]. The aforementioned research showcased the remarkable efficacy of the EO derived from *C. acaulis* for the control of *R. dominica* compared to the EOs tested in the current study [98]. However, in this study, the efficacy of *S. olusatrum* EO significantly surpassed that of *C. acaulis* against *S. oryzae*. In the present investigation, *S. olusatrum* EO treatment completely suppressed *S. oryzae* adults at three days post-exposure at 1000 ppm, as opposed to *C. acaulis*, which suppressed this species after six days of exposure [98]. Nonetheless, achieving rapid and complete suppression of *S. oryzae* adults stands as a significant accomplishment, considering the aforementioned challenges in its control. For instance, in a recent study, combined applications of spinosad with hexaflumuron, lufenuron, and chlorfluazuron on wheat, as well as individual insecticide treatments, resulted in 2.7–56.3% mortalities after seven days of exposure [104]. Additionally, it has been documented that the application of 25 μL/mL of nerolidol or linalool on rice after seven days of exposure resulted in 58.22 and 79.72% *S. oryzae* adult mortality, respectively [105].

Concerning *A. siro*, the investigation into the effectiveness of environmentally friendly pesticides when applied to commodities is scarce. Only a limited number of natural pesticides, such as zeolites, have undergone assessment for *A. siro* control. However, EOs have previously showcased their effectiveness against this pest. As per Kavallieratos et al. [69], when 1000 ppm of *Pimpinella anisum* L. EO-based NE and *T. ammi* EO-based NE were assessed as single and combined treatments on wheat, they resulted in mortality rates ranging from 29.8 to 38.1% for adults and 21.1 to 86.6% for nymphs, observed 7 days after exposure. The findings of this study align with previously observed mortalities of this pest when subjected to green pesticides. Nonetheless, the efficacy of *C. acaulis* EO stood out significantly, achieving mortality rates of 91.1% for adults and 95.6% for nymphs of *A. siro* [98].

Examining various EOs against different pests is imperative due to the diverse outcomes observed, contributing to a more comprehensive pest control strategy. For instance, Kavallieratos et al. [98] demonstrated complete mortality in *T. molitor* larvae using *C. acaulis* EO at 1000 ppm over four days, while the EO showed lower effectiveness for adults. Conversely, in our research, *T. ammi* EO achieved complete mortality in *T. molitor* adults at the same concentration and exposure interval, with no impact on larvae. This combination suggests potential benefits in integrating these EOs for pest management, underscoring the necessity for thorough, multifaceted investigations. This study expands our understanding of the efficacy of *C. maritimum*, *S. olusatrum*, *T. ammi*, and *E. persica* EOs against various developmental stages of nine storage pests. *Smyrnium olusatrum* and *T. ammi* EOs displayed notable effectiveness against a diverse range of arthropod pests, although the performance of each EO varies concerning the targeted species. The mechanism by which EOs affect insect pests is not completely clear. However, their mode of action always relies on the chemical nature and interactions of their major components. Regarding the pesticidal action of *S. olusatrum* EO, its bioactivity could be ascribed to the presence of furanosesquiterpenes, mainly represented by isofuranodiene. This compound has already been reported for its marked pesticidal action on *T. granarium*, *P. truncatus*, *Tetranychus urticae* Koch [68,106], but also on *T. molitor*, *T. castaneum*, and *T. confusum* [88]. Like other molecules containing the furan moiety, isofuranodiene is susceptible to lactonization [107,108], producing five-membered reactive compounds. The latter, under photo-oxidation, can produce toxic radicals lethal to numerous insects and pests [108,109]. In the case of isofuranodiene, its phototoxins could bind, through Michael addition, to the thiol groups of proteins and enzymes such as those involved in the detoxicative functions (e.g., glutathione) [110], with the consequent production of oxidative stress and damage to the tissues. The action of isofuranodiene could be reinforced by that of germacrone and myrcene (20.5 and 29.4% of the EO composition, respectively). Germacrone has been effective against *T. urticae*, *Lasioderma serricorne* (F.), and *T. castaneum* [106,111,112]; while myrcene demonstrated contact toxicity and repellent effects on *T. castaneum* and *Liposcelis bostrychophila* Badonnel [113].

Concerning *T. ammi* EO, its pesticidal potential is strongly correlated with the presence of thymol as a major compound (38.9% of the total composition). Indeed, it was effective against insect vectors such as *Culex pipiens* L. [114] and *Musca domestica* L. [115]. Thymol is reported as a potent inhibitor of acetylcholinesterase (AChE), but also as a molecule interacting with GABA-A and octopamine receptors [114]. In addition to this compound, *p*-cymene (28.0%) and *γ*-terpinene (28.8%) could contribute to the effect registered for *T. ammi* EO and synergize the activity of thymol, as reported in previous research [116,117].

Alternatively, *γ*-terpinene represents one of the major compounds found in *C. maritimum* EO, accounting for 36.1% of the total EO composition. This molecule has exhibited larvicidal activity against various insects, such as *Anopheles anthropophagus* (Xu and Feng), *Aedes aegypti* L., and *Aedes albopictus* Skuse (Diptera: Culicidae) [118,119,120,121,122,123]. It also displayed the ability to suppress the growth and development of *Zeugodacus cucurbitae* (Coquillett) (Diptera, Tephritidae) larvae, compromising the immune system of the insect [124]. As with other monoterpenoids, *γ*-terpinene has been reported to be a competitive inhibitor of acetylcholinesterase (AChE) [125], but the exact mechanism of action is yet to be defined. The pesticidal action of *C. maritimum* EO could be ascribed to the action of *γ*-terpinene, as well as to the synergistic activity of this compound with *β*-phellandrene, dillapiole, thymol methyl ether, and *p*-cymene (14.7, 12.0, 11.2, and 10.1% of the total composition, respectively), all of which have demonstrated insecticidal and pesticidal properties [126,127,128].

Concerning *E. persica* EO, its chemical composition was mainly dominated by *γ*-terpinene (35.8%) and cumin aldehyde (16.6%). In earlier investigations, γ-terpinene demonstrated insecticidal activity, paralleled by cumin aldehyde, which exhibited toxicity against larvae of both *C. pipiens* and *A. albopictus* [129,130]. Therefore, the pesticidal activity of *E. persica* EO could be ascribed to the action of both *γ*-terpinene and cumin aldehyde, but also to the synergism of these compounds with other minor constituents such as *α*-terpinen-7-al and γ- terpinen-7-al.

The wide availability of biomass from *S. olusatrum* and *T. ammi* from spontaneous populations and cultivations, respectively, underscores the potential scalability of these EOs within agrochemical applications. Following the outcomes of the current study, further comprehensive assessments of these EOs are advised. These evaluations should encompass individual and combined analyses to determine their effectiveness across diverse insects, mites, commodities, and concentrations. This approach may facilitate the creation of eco-friendly multi-pesticide formulations suitable for storage environments.

## 4. Materials and Methods

### 4.1. Plant Materials

*Crithmum maritimum* was harvested in 2020 from a cultivation located in the Municipality of Camerano (AN), Italy (N 43°32′08″; E 13°33′03″, 135 m a.s.l., North exposure, with an average incline of 5%). The texture of the soil was silty, with very little presence of sand. The climate at the station is sub-Mediterranean—low-mesotemperate. The cultivation process commenced in 2019 using indigenous seeds. Germination took place in January within a greenhouse, facilitated by a nursery plant tray. Subsequently, transplantation occurred in agricultural land in late March. The cultivation was organic, with no fertilization or irrigation. The plants were not provided with any growing operations or plant protection. Full irradiation was ensured, avoiding the presence of natural weeds through manual operation until the plant reached full-grown habitus to cover its surroundings. Manual seed harvesting occurred in October 2020, coinciding with the full maturation of the seed phase, characterized by the desiccation of the floral structures (umbellifers). Upon collection, the seeds were stored in the farm owner’s warehouse, ensuring strict adherence to hygienic, food-related, and GACP (Good Agricultural and Collection Practices) standards. The ripened seeds (schizocarps) of wild *T. ammi* were collected from Ardabil Province (Iran, N 38°180′; E 48°190′; 1346 m a.s.l.) in August 2021. *Elwendia persica* seeds were harvested from a spontaneous accession in the Binaloud mountains (Iran, N 36°12′; E 50°06′, 2053 m a.s.l.) in the province of Khorasan Razavi, at the full ripening stage in June 2020. The plant locations were specified at the flowering stage and, after identification, voucher specimens (codex no 4517 and 4512, respectively) were deposited in the Herbarium of the Department of Horticultural Science, University of Maragheh, Iran. *Smyrnium olusatrum* flowers were collected in April 2020 during its blooming period from San Severino Marche, central Italy (N 43°13′44.9″; E 13°10′29.1″, 236 m a.s.l.); a voucher specimen (codex: CAME #29339) was stored in the Herbarium Camerinensis, c/o School of Bioscience and Veterinary Medicine, University of Camerino, Italy.

### 4.2. Isolation of EOs

The EOs of the four plant species tested here were obtained through hydro-distillation using a Cleavenger-type apparatus placed on top of a 10 L round flask. A mantle system Falc MA (Falc Instruments, Treviglio, Italy) was employed for heating. Yields of 4.45 and 1.79% (*w*/*w*) were obtained from the distillation of 0.6 kg of *T. ammi* and *C. marithimum* schizocarps, respectively, in 6 L of deionized water. Concerning *E. persica*, 0.820 kg of plant seeds were distilled using 7 L of distilled water, and the EO was isolated with a yield of 3.72% (*w*/*w*). Regarding *S. olusatrum*, 2.2 kg of fresh material were placed in 6 L of deionized water, and a yield of 0.90% *w/dry weight (dw)* was achieved. The *dw* of the plant material was estimated by determining the water content (81.80%) by drying the fresh flowers in an oven at 110 °C for 16 h.

### 4.3. Chemical Analysis of EOs

The chemical analysis of the EOs was performed employing an Agilent 6890 N gas chromatograph furnished with a single quadrupole 5973N mass spectrometer and an auto-sampler 7863 (Agilent, Wilmington, DE, USA). The separation of the EOs’ constituents was achieved through an HP-5MS capillary column (30 m length, 0.25 mm i.d., 0.1 μm film thickness; 5% phenylmethylpolysiloxane) supplied by Agilent (Folsom, CA, USA). The EOs were diluted 1:100 in *n*-hexane before the analysis. The analytical conditions and the interpretation of the chromatograms were in line with those previously reported [131].

### 4.4. Insect and Mite Species

The colonies were maintained in complete darkness at the Laboratory of Agricultural Zoology and Entomology, affiliated with the Agricultural University of Athens. *Oryzaephilus surinamensis* was reared using a blend composed of rolled oats, fragmented brewer’s yeast, and wheat in a ratio of 5 parts oats: 1 part yeast: 5 parts wheat. *Alphitobius diaperinus* were sustained on a diet consisting of yeast and wheat bran in a 1:3 ratio, supplemented with apple cubes for added moisture. *Rhyzopertha dominica*, *T. granarium*, and *S. oryzae* were exclusively maintained on intact wheat grains. *Tribolium* species were cultivated on a diet composed of wheat flour supplemented with an extra 5% of brewer’s yeast. *Tenebrio molitor* received oat bran and slices of potato to enhance moisture levels. *Acarus siro* were maintained on a diet comprising wheat germ, brewer’s yeast, and oat flakes in a 10:1:10 ratio. For the insect species, the temperature was set at 30 °C with a relative humidity (RH) of 65%, while for the mite species, the conditions were 25 °C at 80% RH. The involved insect participants were of indeterminate sex and under 14 days old for adult *T. molitor*, *Tribolium* spp., *S. oryzae*, *R. dominica*, and *O. surinamensis*, younger than 24 h for *T. granarium* adults, or less than one week old for *A. diaperinus* adults. Regarding the larvae used in the experiments, they ranged from 3rd to 4th instar for the *Tribolium* species and *O. surinamensis*, measured between 1 to 1.4 cm in length for *T. molitor*, were of medium size (2–4 mm) for *T. granarium*, or were smaller than 0.7 cm in length for *A. diaperinus*. The *A. siro* specimens were of unidentified sex. They were acquired from colonies ranging in age between 1 and 21 days, while identification of nymphs and adults was based on external morphology, particularly discerning shorter body setae in the former [132].

### 4.5. Grains

For the mortality bioassays, uncontaminated hard wheat, *Triticum durum* Desf. (var. Claudio), devoid of pesticides and infestations, was utilized. Grain moisture was calculated to be 13.2% implementing a moisture meter (mini-GAC plus, Dickey-John Europe S.A.S., Colombes, France) before testing [98].

### 4.6. Bioassays

The bioassays commenced consequent to the conduction of preliminary trials that evaluated two concentrations: 500 and 1000 ppm, representing 500 and 1000 μL EO of each EO per kg of wheat, respectively. The test solutions of EOs were formulated by first dissolving 125 and 250 μL of each EO in pure ethanol at a ratio of 1:1 (*v*/*v*) for 500 and 1000 ppm, respectively. Subsequently, the mixture was adjusted to reach a total volume of 750 μL using Tween 80 in a 0.3% (*v*/*v*) aqueous solution [98]. The application of EO solutions was conducted by evenly spraying 0.25 kg of wheat separately on trays via the BD-134 K airbrush (Fengda^®^, London, UK), with each tray representing a treatment replicate. Supplementary solutions, comprising (i) water, (ii) 99.8% ethanol, (iii) carrier control (Tween 80 in combination with ethanol and water), and (iv) a positive control, i.e., Actellic EC containing the active component pirimiphos–methyl (50%) at the concentration appearing on the pesticide label (5 ppm = 5 μL/kg wheat), were administered to additional lots for control purposes. Each of these solutions was applied using distinct airbrushes. The treated amounts of wheat were transferred into glass containers with a capacity of 1 L, where a manual agitation lasting 10 min was meticulously performed. This was undertaken to guarantee the uniform dispersion of both the EO solutions and controls throughout the wheat kernels. Following this, three sub-samples of either 10 g or 1 g of wheat, designated for insects and mites, respectively, were extracted from every treated 1 L container of wheat (EO or control) using individual spoons. These wheat sub-samples were precisely weighed on filter paper using the Precisa XB3200D electronic balance (Alpha Analytical Instruments, Athens, Greece). Distinct filter paper was utilized between each weighing. Subsequently, the 10 g and 1 g samples were conveyed into glass vials selected specifically for the insect species (125 mm in height and 75 mm in diameter) or the mite species (60 mm in height and 27 mm in diameter). To facilitate air circulation within the larger vials, their caps were equipped with a 15 mm in diameter opening sealed with fabric, whereas the smaller vials were fitted with caps featuring perforations [99,132].

The interior walls near the vial caps were coated with a 60 wt% by weight dispersion of polytetrafluoroethylene (Sigma-Aldrich Chemie GmbH, Taufkirchen, Germany) in water to secure the arthropods inside. Arthropod species were individually introduced in groups of 10 adults, larvae, or nymphs into the designated vials. The vials were then placed in incubators set at 30 °C and 65% RH for *S. oryzae*, *R. dominica*, *O. surinamensis*, *T. molitor*, *A. diaperinus*, and the *Tribolium* species, and at 25 °C and 80% RH specifically for *A. siro* throughout the experimental duration [131]. To prevent cannibalism among *A. diaperinus* individuals, every sub-replication comprised 10 vials, each housing a single arthropod [132]. After exposure intervals from 1 to 7 days, mortality assessments were conducted using the Olympus SZX9 stereomicroscope (Bacacos S.A., Athens, Greece). Dead specimens were carefully examined with individual fine brushes for each concentration of controls or EOs, ensuring that any movement from the arthropods was detected. This entire process was repeated three times, comprising three replications and three sub-replications, incorporating new sets of wheat, arthropods, and glass vials for each iteration.

### 4.7. Data Analysis

Control mortality, staying below 5%, required no adjustment. To standardize variance, the dataset was log (x + 1) transformed before analysis [133,134]. Data on each arthropod species underwent analysis using a repeated measures model, with exposure as the repeated factor and mortality as the response variable. The main effects considered were EO type and concentration [135]. All analyses were executed using JMP 16.2 software [136]. For mean separation, the Tukey–Kramer HSD test at a 0.05 significance level was applied [137].

## Figures and Tables

**Table 1:** Chemical composition of the essential oils derived from *Trachyspermum ammi*, *Crithmum maritimum*, *Smyrnium olusatrum*, and *Elwendia persica*.

RI ^b^	RI Lit ^c^	Component ^a^	*T. ammi*	*C. maritimum*	*S. olusatrum*	*E. persica*	ID ^e^
			Average Area % ^d^				
926	924	*α*-thujene	0.4	0.4	tr ^f^	0.3	RI, MS
929	932	*α*-pinene	0.2	7.0	2.0	0.5	Std
947	946	camphene	—	tr	—	tr	RI, MS
972	969	sabinene	—	5.1	—	0.8	RI, MS
975	974	*β*-pinene	2.2	0.4	0.3	0.8	Std
989	988	myrcene	0.3	0.5	29.4	0.5	Std
1002	1002	*α*-phellandrene	—	0.1	—	0.1	RI, MS
1004	998	n-octanal	—	tr	—	—	RI, MS
1009	1008	*δ*-3-carene	—	tr	0.4	tr	Std
1016	1014	*α*-terpinene	0.4	0.3	—	0.3	RI, MS
1024	1020	*p*-cymene	28.0	10.1	0.1	8.3	Std
1025	1026	1,8-cineole	—	—	—	0.4	Std
1027	1025	*β*-phellandrene	0.4	14.7	15.3	—	RI, MS
1028	1024	limonene	—	—	—	5.9	Std, RI, MS
1036	1032	(Z)-*β*-ocimene	—	0.2	—	—	RI, MS
1048	1044	(*E*)-*β*-ocimene	—	—	1.4	tr	RI, MS
1058	1054	*γ*-terpinene	28.8	36.1	0.2	35.8	Std
1062	1065	(*Z*)-sabinene hydrate	—	0.3	—	—	RI, MS
1084	1086	terpinolene	—	tr	0.1	0.4	Std
1094	1098	(*E*)-sabinene hydrate	—	0.2	—	—	RI, MS
1100	1095	linalool	—	tr	—	tr	Std
1117	1119	(*E*)- *p*-mentha-2,8-dien-1-ol	—	tr	—	—	RI, MS
1122	1122	*α*-campholenal	—	tr	—	—	RI, MS
1176	1174	terpinen-4-ol	0.1	0.6	—	0.7	Std
1183	1179	*p*-cymen-8-ol	—	—	—	tr	RI, MS
1189	1196	*p*-menth-3-en-7-al	—	—	—	2.0	RI, MS
1190	1186	*α*-terpineol	tr	0.1	—	—	Std
1233	1232	thymol, methyl ether	—	11.2	—	—	RI, MS
1235	1238	cumin aldehyde	—	—	—	16.6	RI, MS
1242	1241	carvacrol, methyl ether	—	0.0	—	—	RI, MS
1277	1283	*α*-terpinen-7-al	—	—	—	11.7	RI, MS
1280	1287	bornyl acetate	—	tr	—	0.3	Std
1285	1290	*γ*-terpinen-7-al	—	—	—	14.0	RI, MS
1292	1289	thymol	38.9	0.2	—	—	Std
1302	1298	carvacrol	0.2	tr	—	—	Std
1394	1389	*β*-elemene ^g^	—	—	0.1	—	RI, MS
1434	1434	*γ*-elemene ^g^	—	—	tr	—	RI, MS
1484	1484	germacrene D	—	—	0.6	—	Std, RI, MS
1499	1499	curzerene ^g^	—	—	16.3	—	RI, MS
1558	1555	elemicin	—	tr	—	—	RI, MS
1561	1559	germacrene B ^g^	—	—	2.0	—	RI, MS
1607	1602	(*E*)-*β*-elemenone ^g^	—	—	0.3	—	RI, MS
1626	1622	dillapiole	—	12.0	—	—	RI, MS
1693	1688	isofuranodiene ^g^	—	—	6.2	—	Std, RI, MS
1696	1693	germacrene	—	—	20.5	—	RI, MS
1841	1833	furano-4(15)-eudesmen-1-one ^g^	—	—	0.4	—	RI, MS
18884	1879	furanoeremophil-1-one ^g^	—	—	4.0	—	RI, MS
1992	1988	1-*β*-acetoxyfurano-4(15)-eudesmene ^g^	—	—	0.1	—	RI, MS
		Monoterpene hydrocarbons	60.7	75.0	49.2	53.6	
		Oxygenated monoterpenes	39.2	12.8	0.0	45.4	
		Sesquiterpenes hydrocarbons	0.0	0.0	2.7	0.0	
		Oxygenated sesquiterpenes	0.0	0.0	20.8	0.0	
		Furanosesquiterpenes	0.0	0.0	27.0	0.0	
		Phenylpropanoids	0.0	12.0	0.0	0.0	
		Others	0.0	0.0	0.0	0.3	
		Total identified (%)	99.9	99.7	99.7	99.3	

^a^ Compounds are reported depending on the order of their elution from an HP-5MS capillary column. ^b^ Linear retention index experimentally obtained through the analysis of a homologous series of C_7_–C_30_ alkanes on HP-5MS capillary column. ^c^ Linear retention index reported in Adams (2007) [Identification of Essential Oil Components by Gas Chromatography/Mass Spectrometry; Allured Publishing Corp., Carol Stream, IL, USA]. ^d^ Relative area percentage values are means of two independent analyses, showing RSD% values < 20% in all cases. ^e^ Identification methods: Std, derived from the comparison with pure standards; MS, derived from comparison with WILEY, ADAMS, and NIST 08 MS libraries; RI, based on comparison of RI with those found in ADAMS, FFNSC2, and NIST 08 libraries. ^f^ tr, % < 0.05. ^g^ Results of the quantitative analysis are altered due to thermal degradation phenomena.

**Table 2:** MANOVA parameters depicting the main effects and their interactions leading to the observed mortalities of *Alphitobius diaperinus*, *Tribolium castaneum, T. confusum*, *Tenebrio molitor*, *Trogoderma granarium*, and *Oryzaephilus surinamensis* larvae and adults, *Rhyzopertha dominica* and *Sitophilus oryzae* adults, and *Acarus siro* nymphs and adults, between and within exposures (error df = 80 for all species and developmental stages).

		Between Exposures				Within Exposures			
Pest Species		Intercept	Concentration	EO Type	Concentration × EO Type	Exposure	Exposure × Concentration	Exposure × EO Type	Exposure × Concentration × EO Type
	df	1	1	4	4	6	6	24	24
*A. diaperinus* larvae	*F*	2999.8	35.2	681.5	16.1	87.5	2.3	12.4	2.5
	*p*	<0.01	<0.01	<0.01	<0.01	<0.01	0.04	<0.01	<0.01
*A. diaperinus* adults	*F*	126.7	27.5	34.7	8.4	36.9	5.3	8.1	1.6
	*p*	<0.01	<0.01	<0.01	<0.01	<0.01	<0.01	<0.01	0.03
*T. castaneum* larvae	*F*	13,484.4	144.8	37.5	12.9	128.0	10.9	8.9	2.9
	*p*	<0.01	<0.01	<0.01	<0.01	<0.01	<0.01	<0.01	<0.01
*T. castaneum* adults	*F*	237.8	120.3	87.3	27.3	274.1	254.2	33.5	33.3
	*p*	<0.01	<0.01	<0.01	<0.01	<0.01	<0.01	<0.01	<0.01
*T. confusum* larvae	*F*	11,897.9	95.8	58.6	15.9	137.8	6.9	7.1	3.8
	*p*	<0.01	<0.01	<0.01	<0.01	<0.01	<0.01	<0.01	<0.01
*T. confusum* adults	*F*	50.1	24.0	7.1	2.2	14.3	8.7	4.6	3.1
	*p*	<0.01	<0.01	<0.01	0.08	<0.01	<0.01	<0.01	<0.01
*T. molitor* larvae	*F*	235.0	9.9	75.9	2.9	37.6	1.3	5.7	1.1
	*p*	<0.01	0.01	<0.01	0.03	<0.01	0.29	<0.01	0.39
*T. molitor* adults	*F*	2847.3	30.1	284.8	5.8	91.8	0.8	11.6	2.1
	*p*	<0.01	<0.01	<0.01	<0.01	<0.01	0.55	<0.01	<0.01
*T. granarium* larvae	*F*	1444.9	41.0	186.8	6.5	53.8	2.0	4.4	2.0
	*p*	<0.01	<0.01	<0.01	<0.01	<0.01	0.08	<0.01	<0.01
*T. granarium* adults	*F*	8020.9	42.9	35.3	7.3	200.3	4.4	9.2	4.4
	*p*	<0.01	<0.01	<0.01	0.01	<0.01	<0.01	<0.01	<0.01
*O. surinamensis* larvae	*F*	5089.8	209.9	98.2	32.2	108.9	2.7	6.6	2.1
	*p*	<0.01	<0.01	<0.01	<0.01	<0.01	0.02	<0.01	<0.01
*O. surinamensis* adults	*F*	2234.6	19.1	48.6	3.8	74.9	0.5	5.3	1.2
	*p*	<0.01	<0.01	<0.01	<0.01	<0.01	0.77	<0.01	0.27
*R. dominica* adults	*F*	3136.6	185.2	41.2	33.7	58.2	2.1	4.1	2.1
	*p*	<0.01	<0.01	<0.01	<0.01	<0.01	0.07	<0.01	<0.01
*S. oryzae* adults	*F*	2511.0	37.4	242.1	7.7	35.0	1.9	4.3	1.6
	*p*	<0.01	<0.01	<0.01	<0.01	<0.01	0.09	<0.01	0.03
*A. siro* nymphs	*F*	812.0	12.1	23.3	1.9	114.5	1.7	3.3	0.8
	*p*	<0.01	<0.01	<0.01	0.12	<0.01	0.13	<0.01	0.70
*A. siro* adults	*F*	1128.9	3.5	20.7	0.5	130.1	0.5	2.5	0.5
	*p*	<0.01	0.07	<0.01	0.77	<0.01	0.81	<0.01	0.99

**Table 3:** Mean (%) mortality ± standard errors (SEs) of *Alphitobius diaperinus* larvae and adults after 1–7 days in wheat treated with *Crithmum maritimum*, *Smyrnium olusatrum*, *Trachyspermum ammi*, and *Elwendia persica* essential oils (EOs) at two concentrations, with positive control, pirimiphos–methyl.

Exposure	1 Day	2 Days	3 Days	4 Days	5 Days	6 Days	7 Days		
				Larvae					
EO Type	Concentration:	500 ppm						*F*	*p*
*Crithmum maritimum*	0.0 ± 0.0 Bc	0.0 ± 0.0 Bc	0.0 ± 0.0 Bc	0.0 ± 0.0 Bc	3.3 ± 1.7 Bb	11.1 ± 2.0 Ac	13.3 ± 1.7 Ac	35.9	<0.01
*Smyrnium olusatrum*	28.9 ± 3.5 Ca	43.3 ± 4.1 BCa	51.1 ± 5.4 ABa	53.3 ± 4.7 ABa	57.8 ± 5.7 ABa	66.7 ± 6.2 ABa	68.9 ± 6.1 Aa	8.6	<0.01
pirimiphos-methyl	8.9 ± 2.0 Cb	15.6 ± 2.4 Bb	21.1 ± 3.1 ABb	26.7 ± 3.3 ABb	28.9 ± 3.5 ABa	34.4 ± 4.8 Ab	36.7 ± 4.7 Ab	12.5	<0.01
*F*	72.5	600.0	698.8	1093.0	101.6	170.7	620.0		
*p*	<0.01	<0.01	<0.01	<0.01	<0.01	<0.01	<0.01		
	Concentration:	1000 ppm							
*Crithmum maritimum*	0.0 ± 0.0 Cc	5.6 ± 1.7 BCc	8.9 ± 2.6 Bc	15.6 ± 3.7 ABc	18.9 ± 5.1 ABc	32.2 ± 6.0 Ab	44.4 ± 4.8 Ab	13.1	<0.01
*Smyrnium olusatrum*	45.6 ± 3.4 Da	72.2 ± 4.7 Ca	77.8 ± 4.7 BCa	92.2 ± 3.6 ABa	98.9 ± 1.1 Aa	100.0 ± 0.0 Aa	100.0 ± 0.0 Aa	35.7	<0.01
pirimiphos-methyl	8.9 ± 1.1 Db	14.4 ± 2.4 CDb	20.0 ± 2.4 BCb	23.3 ± 1.7 ABCb	27.8 ± 2.2 ABb	33.3 ± 2.9 ABb	37.8 ± 3.2 Ab	14.6	<0.01
*F*	197.2	83.9	82.8	93.6	86.8	557.6	1287.0		
*p*	<0.01	<0.01	<0.01	<0.01	<0.01	<0.01	<0.01		
				**Adults**					
	Concentration:	1000 ppm							
*Crithmum maritimum*	0.0 ± 0.0 B	0.0 ± 0.0 B	10.0 ± 2.9 ABa	13.3 ± 4.4 Aa	13.3 ± 4.4 Aa	13.3 ± 4.4 Aab	13.3 ± 4.4 Ab	4.9	<0.04
*Elwendia persica*	0.0 ± 0.0 B	0.0 ± 0.0 B	4.4 ± 1.8 ABb	5.6 ± 1.8 ABb	5.6 ± 1.8 ABb	6.7 ± 1.7 Ab	6.7 ± 1.7 Ab	4.0	<0.02
pirimiphos-methyl	0.0 ± 0.0 D	0.0 ± 0.0 D	4.4 ± 1.8 CDb	7.8 ± 2.2 BCb	16.7 ± 2.9 ABa	22.2 ± 2.2 Aa	23.3 ± 2.4 Aa	25.8	<0.01
*F*	-	-	5.4	7.0	12.2	20.7	21.1		
*p*	-	-	0.01	0.01	<0.01	<0.01	<0.01		

For each EO, within each row, means followed by the same uppercase letter are not significantly different (df = 6, 62; Tukey HSD test at *p* = 0.05). For each concentration, within each column, means followed by the same lowercase letter are not significantly different (df = 4, 44; Tukey HSD test at *p* = 0.05). No significant differences were recorded where no letters exist. No statistical analysis was performed where dashes exist. Due to zero values, mortality data for adults exposed to any EO at 500 ppm are not shown. Due to zero values, mortality data for adults exposed to *S. lustratum* and *T. ammi* EOs at 1000 ppm are not shown.

**Table 4:** Mean (%) mortality ± standard errors (SE) of *Tribolium castaneum* larvae and adults after 1–7 days in wheat treated with *Crithmum maritimum*, *Smyrnium olusatrum*, *Trachyspermum ammi*, and *Elwendia persica* essential oils (EOs) at two concentrations, with positive control, pirimiphos–methyl.

Exposure	1 Day	2 Days	3 Days	4 Days	5 Days	6 Days	7 Days		
				Larvae					
EO Type	Concentration:	500 ppm						*F*	*p*
*Crithmum maritimum*	0.0 ± 0.0 Dc	0.0 ± 0.0 Dd	13.3 ± 4.1 Cb	23.3 ± 2.4 Bb	42.2 ± 4.0 ABb	61.1 ± 5.6 Aab	67.8 ± 6.2 Aab	78.8	<0.01
*Smyrnium olusatrum*	17.8 ± 3.6 Ca	44.4 ± 2.4 Ba	55.6 ± 3.4 ABa	68.9 ± 5.4 Aa	73.3 ± 4.7 Aa	76.7 ± 3.7 Aa	77.8 ± 3.2 Aa	40.1	<0.01
*Trachyspermum ammi*	10.0 ± 2.9 Cab	16.7 ± 3.3 BCbc	28.9 ± 3.9 ABa	34.4 ± 5.0 ABb	51.1 ± 3.9 Ab	57.8 ± 4.9 Aab	61.1 ± 4.2 Aabc	12.4	<0.01
*Elwendia persica*	3.3 ± 1.7 Cbc	8.9 ± 2.0 Bc	23.3 ± 4.1 Aa	35.6 ± 3.8 Ab	38.9 ± 4.2 Ab	44.4 ± 5.0 Ab	48.9 ± 3.5 Ac	24.9	<0.01
pirimiphos-methyl	20.0 ± 3.3 Ca	22.2 ± 3.6 Cab	28.9 ± 3.5 BCa	35.6 ± 3.4 ABb	41.1 ± 3.1 ABb	51.1 ± 4.2 Ab	56.7 ± 2.9 Abc	12.7	<0.01
*F*	18.9	35.1	8.2	11.2	9.3	5.4	6.2		
*p*	<0.01	<0.01	<0.01	<0.01	<0.01	0.01	0.01		
	Concentration:	1000 ppm							
*Crithmum maritimum*	12.2 ± 2.8 Bc	16.7 ± 2.9 Bd	40.0 ± 6.0 Abc	61.1 ± 3.5 Ab	82.2 ± 4.3 Ab	91.1 ± 3.9 Aab	91.1 ± 3.9 Aa	19.2	<0.01
*Smyrnium olusatrum*	48.9 ± 3.9 Ca	78.9 ± 5.6 Ba	91.1 ± 4.6 ABa	100.0 ± 0.0 Aa	100.0 ± 0.0 Aa	100.0 ± 0.0 Aa	100.0 ± 0.0 Aa	34.3	<0.01
*Trachyspermum ammi*	28.9 ± 2.0 Dab	52.2 ± 3.2 Cab	77.8 ± 2.8 Ba	93.3 ± 3.3 Aa	100.0 ± 0.0 Aa	100.0 ± 0.0 Aa	100.0 ± 0.0 Aa	134.9	<0.01
*Elwendia persica*	22.2 ± 2.8 Dab	33.3 ± 3.3 Cbc	51.1 ± 4.8 Bb	65.7 ± 4.8 ABb	73.3 ± 5.8 ABb	81.1 ± 5.1 Ab	90.0 ± 4.4 Aa	31.3	<0.01
pirimiphos-methyl	17.8 ± 2.8 Ebc	22.2 ± 3.2 DEcd	27.8 ± 2.2 CDc	34.4 ± 1.8 BCc	42.2 ± 1.5 ABc	53.3 ± 2.9 Ac	58.9 ± 3.1 Ab	25.4	<0.01
*F*	9.0	15.1	21.3	<67.5	59.9	38.7	33.7		
*p*	<0.01	<0.01	<0.01	0.01	<0.01	<0.01	<0.01		
				**Adults**					
	Concentration:	500 ppm							
*Smyrnium olusatrum*	0.0 ± 0.0 B	0.0 ± 0.0 B	2.2 ± 1.5 AB	5.6 ± 2.4 ABa	7.8 ± 3.6 ABa	10.0 ± 3.7 ABa	13.3 ± 3.3 Aa	4.6	<0.01
pirimiphos-methyl	0.0 ± 0.0	0.0 ± 0.0	0.0 ± 0.0	0.0 ± 0.0 b	0.0 ± 0.0 b	0.0 ± 0.0 b	0.0 ± 0.0 b	-	-
*F*	-	-	2.3	6.3	6.1	9.6	25.8		
*p*	-	-	0.08	0.01	0.01	<0.01	<0.01		
	Concentration:	1000 ppm							
*Crithmum maritimum*	0.0 ± 0.0 B	0.0 ± 0.0 Bb	0.0 ± 0.0 Bb	0.0 ± 0.0 Bc	1.1 ± 1.1 ABc	2.2 ± 1.5 ABcd	5.6 ± 2.4 Ac	3.2	<0.01
*Smyrnium olusatrum*	0.0 ± 0.0 E	26.7 ± 2.4 Da	46.7 ± 5.0 Ca	58.9 ± 5.4 BCa	71.1 ± 3.5 ABa	77.8 ± 2.8 ABa	81.1 ± 2.6 Aa	457.0	<0.01
*Trachyspermum ammi*	0.0 ± 0.0 B	0.0 ± 0.0 Bb	1.1 ± 1.1 ABb	2.2 ± 1.5 ABc	4.4 ± 1.8 Abc	5.6 ± 2.4 Ac	5.6 ± 2.4 Ac	2.4	0.04
*Elwendia persica*	0.0 ± 0.0 C	0.0 ± 0.0 Cb	3.3 ± 1.7 BCb	6.7 ± 1.7 ABb	7.8 ± 1.5 ABb	13.3 ± 1.7 Ab	13.3 ± 1.7 Ab	18.7	<0.01
pirimiphos-methyl	0.0 ± 0.0	0.0 ± 0.0 b	0.0 ± 0.0 b	0.0 ± 0.0 c	0.0 ± 0.0 c	0.0 ± 0.0 d	0.0 ± 0.0 c	-	-
*F*	-	1550.2	53.2	49.3	39.1	45.4	33.9		
*p*	-	<0.01	<0.01	<0.01	<0.01	<0.01	<0.01		

For each EO, within each row, means followed by the same uppercase letter are not significantly different (df = 6, 62; Tukey HSD test at *p* = 0.05). For each concentration, within each column, means followed by the same lowercase letter are not significantly different (df = 4, 44; Tukey HSD test at *p* = 0.05). No significant differences were recorded where no letters exist. No statistical analysis was performed where dashes exist. Due to zero values, mortality data for adults exposed to *C. maritimum*, *T. ammi*, and *E. persica* EOs at 500 ppm are not shown.

**Table 5:** Mean (%) mortality ± standard errors (SE) of *Tribolium confusum* larvae and adults after 1–7 days in wheat treated with *Crithmum maritimum*, *Smyrnium olusatrum*, *Trachyspermum ammi*, and *Elwendia persica* essential oils (EOs) at two concentrations, with positive control, pirimiphos–methyl.

Exposure	1 Day	2 Days	3 Days	4 Days	5 Days	6 Days	7 Days		
				Larvae					
EO Type	Concentration:	500 ppm						*F*	*p*
*Crithmum maritimum*	0.0 ± 0.0 Ed	3.3 ± 1.7 DEc	5.6 ± 1.8 CDc	12.2 ± 2.2 BCb	16.7 ± 2.4 ABc	22.2 ± 3.6 ABc	36.7 ± 5.0 Ac	23.5	<0.01
*Smyrnium olusatrum*	33.3 ± 4.7 Ba	51.1 ± 4.8 Aa	57.8 ± 5.5 Aa	61.1 ± 4.8 Aa	62.2 ± 4.0 Aa	65.6 ± 3.4 Aa	65.6 ± 3.4 Aab	6.3	<0.01
*Trachyspermum ammi*	25.6 ± 3.8 Cab	41.1 ± 6.1 BCab	47.8 ± 5.7 ABCab	52.2 ± 6.6 ABa	64.4 ± 3.4 ABa	75.6 ± 4.4 Aa	80.0 ± 4.1 Aa	7.9	<0.01
*Elwendia persica*	6.7 ± 1.7 Dc	15.6 ± 2.6 Cb	20.0 ± 3.3 BCb	32.2 ± 2.2 ABa	36.7 ± 2.4 ABb	41.1 ± 3.9 ABb	50.0 ± 1.7 Ab	20.0	<0.01
pirimiphos-methyl	14.4 ± 3.8 Cbc	24.4 ± 2.9 Bab	32.2 ± 3.2 ABab	43.3 ± 2.9 ABa	55.6 ± 2.4 Aa	62.2 ± 3.2 Aa	67.8 ± 2.8 Aab	14.7	<0.01
*F*	23.0	28.2	21.8	15.2	53.4	29.1	19.0		
*p*	<0.01	<0.01	<0.01	<0.01	<0.01	<0.01	<0.01		
	Concentration:	1000 ppm							
*Crithmum maritimum*	12.2 ± 3.2 Ccd	24.4 ± 3.8 Bb	37.8 ± 4.9 ABbc	48.9 ± 6.1 ABb	63.3 ± 6.5 Abc	76.7 ± 8.0 Abc	82.2 ± 7.6 Abc	15.0	<0.01
*Smyrnium olusatrum*	38.9 ± 3.5 Cab	73.3 ± 3.3 Ba	81.1 ± 2.6 ABa	86.7 ± 3.3 ABa	87.8 ± 3.2 ABa	90.0 ± 2.9 ABab	96.7 ± 1.7 Aab	41.0	<0.01
*Trachyspermum ammi*	60.0 ± 6.9 Ca	75.6 ± 5.3 Ba	86.7 ± 4.1 ABa	100.0 ± 0.0 Aa	100.0 ± 0.0 Aa	100.0 ± 0.0 Aa	100.0 ± 0.0 Aa	18.8	<0.01
*Elwendia persica*	6.7 ± 1.7 Cd	24.4 ± 1.8 Bb	47.8 ± 2.2 ABb	60.0 ± 3.7 Ab	81.1 ± 5.6 Aab	87.8 ± 6.0 Aab	94.4 ± 2.9 Aab	41.9	<0.01
pirimiphos-methyl	16.7 ± 2.2 Ebc	25.6 ± 2.4 Db	31.1 ± 2.6 CDc	43.3 ± 3.3 BCb	57.8 ± 4.5 ABc	64.4 ± 3.4 Ac	68.9 ± 2.0 Ac	37.6	<0.01
*F*	13.9	40.5	25.3	19.7	10.6	6.7	9.8		
*p*	<0.01	<0.01	<0.01	<0.01	<0.01	<0.01	<0.01		
				**Adults**					
	Concentration:	500 ppm							
*Elwendia persica*	0.0 ± 0.0	0.0 ± 0.0	2.2 ± 1.5	3.3 ± 1.7 a	3.3 ± 1.7 a	4.4 ± 1.8 a	4.4 ± 1.8 a	1.8	0.11
pirimiphos-methyl	0.0 ± 0.0	0.0 ± 0.0	0.0 ± 0.0	0.0 ± 0.0 b	0.0 ± 0.0 b	0.0 ± 0.0 b	0.0 ± 0.0 b	-	-
*F*	-	-	2.3	4.0	4.0	6.4	6.4		
*p*	-	-	0.08	0.01	0.01	0.01	0.01		
	Concentration:	1000 ppm							
*Crithmum maritimum*	2.2 ± 1.5	2.2 ± 1.5	2.2 ± 1.5 ab	2.2 ± 1.5 ab	5.6 ± 1.8 a	5.6 ± 1.8 ab	7.8 ± 2.2 a	1.7	0.14
*Smyrnium olusatrum*	0.0 ± 0.0 B	0.0 ± 0.0 B	0.0 ± 0.0 Bb	4.4 ± 1.8 ABab	5.6 ± 1.8 ABa	8.9 ± 3.1 Aa	10.0 ± 2.9 Aa	5.5	<0.01
*Trachyspermum ammi*	0.0 ± 0.0 B	0.0 ± 0.0 B	0.0 ± 0.0 Bb	0.0 ± 0.0 Bb	0.0 ± 0.0 Bb	2.2 ± 1.5 Bab	13.3 ± 2.9 Aa	24.2	<0.01
*Elwendia persica*	0.0 ± 0.0 B	0.0 ± 0.0 B	5.6 ± 1.8 ABa	6.7 ± 1.7 Aa	7.8 ± 2.2 Aa	7.8 ± 2.2 Aa	8.9 ± 2.0 Aa	5.6	<0.01
pirimiphos-methyl	0.0 ± 0.0	0.0 ± 0.0	0.0 ± 0.0 b	0.0 ± 0.0 b	0.0 ± 0.0 b	0.0 ± 0.0 b	0.0 ± 0.0 b	-	-
*F*	2.3	2.3	5.7	5.2	6.0	3.6	6.5		
*p*	0.08	0.08	0.01	0.01	0.01	0.01	0.01		

For each EO, within each row, means followed by the same uppercase letter are not significantly different (df = 6, 62; Tukey HSD test at *p* = 0.05). For each concentration, within each column, means followed by the same lowercase letter are not significantly different (df = 4, 44; Tukey HSD test at *p* = 0.05). No significant differences were recorded where no letters exist. No statistical analysis was performed where dashes exist. Due to zero values, mortality data for adults exposed to *C. maritimum*, *S. olustratum*, and *T. ammi* EOs at 500 ppm are not shown.

**Table 6:** Mean (%) mortality ± standard errors (SE) of *Tenebrio molitor* larvae and adults after 1–7 days in wheat treated with *Crithmum maritimum*, *Smyrnium olusatrum*, *Trachyspermum ammi*, and *Elwendia persica* essential oils (EOs) at two concentrations, with positive control, pirimiphos–methyl.

Exposure	1 Day	2 Days	3 Days	4 Days	5 Days	6 Days	7 Days		
				Larvae					
EO Type	Concentration:	500 ppm						*F*	*p*
*Smyrnium olusatrum*	0.0 ± 0.0 B	0.0 ± 0.0 Bb	0.0 ± 0.0 Bb	3.3 ± 1.7 ABb	4.4 ± 1.8 ABb	7.8 ± 2.8 Ab	11.1 ± 2.6 Ab	6.6	<0.01
pirimiphos-methyl	1.1 ± 1.1 D	4.4 ± 2.4 CDa	7.8 ± 2.2 BCa	11.1 ± 2.0 ABa	13.3 ± 1.7 ABa	20.0 ± 2.9 Aa	30.0 ± 2.4 Aa	15.7	<0.01
*F*	1.0	3.9	15.6	19.7	34.8	35.4	72.6		
*p*	0.42	0.01	<0.01	<0.01	<0.01	<0.01	<0.01		
	Concentration:	1000 ppm							
*Crithmum maritimum*	0.0 ± 0.0 B	0.0 ± 0.0 Bb	0.0 ± 0.0 Bb	1.1 ± 1.1 ABb	2.2 ± 1.5 ABb	3.3 ± 1.7 ABb	4.4 ± 1.8 Ab	2.4	0.04
*Smyrnium olusatrum*	0.0 ± 0.0 C	2.2 ± 1.5 Cab	5.6 ± 1.8 BCa	10.0 ± 2.4 ABa	12.2 ± 3.2 ABa	15.6 ± 3.8 ABa	18.9 ± 3.1 Aa	10.1	<0.01
*Elwendia persica*	0.0 ± 0.0	0.0 ± 0.0 b	0.0 ± 0.0 b	0.0 ± 0.0 b	1.1 ± 1.1 b	1.1 ± 1.1 b	2.2 ± 1.5 bc	1.2	0.34
pirimiphos-methyl	2.2 ± 1.5 D	5.6 ± 1.8 CDa	6.7 ± 1.7 BCDa	8.9 ± 1.1 ABCa	14.4 ± 3.4 ABCa	18.9 ± 2.6 ABa	31.1 ± 1.1 Aa	10.3	<0.01
*F*	2.3	5.7	9.7	20.2	13.0	23.8	35.4		
*p*	0.08	0.01	<0.01	<0.01	<0.01	<0.01	<0.01		
				**Adults**					
	Concentration:	500 ppm							
*Smyrnium olusatrum*	2.2 ± 1.5 Bb	4.4 ± 2.4 Bc	7.8 ± 2.2 Bc	26.7 ± 4.4 Ab	40.0 ± 3.7 Ab	67.8 ± 4.7 Ab	75.6 ± 2.9 Ab	26.5	<0.01
*Trachyspermum ammi*	13.3 ± 3.3 Ca	41.1 ± 7.2 Ba	64.4 ± 6.3 ABa	87.8 ± 4.7 Aa	96.9 ± 1.7 Aa	100.0 ± 0.0 Aa	100.0 ± 0.0 Aa	23.3	<0.01
*Elwendia persica*	0.0 ± 0.0 Bb	0.0 ± 0.0 Bc	1.1 ± 1.1 ABd	2.2 ± 1.5 ABc	6.7 ± 2.4 ABc	6.7 ± 2.4 ABc	7.8 ± 2.2 Ac	4.7	<0.01
pirimiphos-methyl	7.8 ± 1.5 Ea	12.2 ± 2.2 DEb	21.1 ± 2.0 CDb	32.2 ± 1.5 BCb	42.2 ± 2.8 ABb	51.1 ± 2.6 ABb	64.4 ± 2.9 Ab	30.5	<0.01
*F*	12.5	52.7	59.0	67.2	84.9	97.9	112.9		
*p*	<0.01	<0.01	<0.01	<0.01	<0.01	<0.01	<0.01		
	Concentration:	1000 ppm							
*Crithmum maritimum*	0.0 ± 0.0 c	0.0 ± 0.0 c	0.0 ± 0.0 d	0.0 ± 0.0 d	0.0 ± 0.0 d	0.0 ± 0.0 d	1.1 ± 1.1 d	1.0	0.44
*Smyrnium olusatrum*	6.7 ± 1.7 Db	15.6 ± 3.4 CDb	20.0 ± 2.4 BCb	41.1 ± 3.5 ABb	65.6 ± 4.4 Ab	88.9 ± 4.6 Aa	96.7 ± 2.4 Aa	27.7	<0.01
*Trachyspermum ammi*	27.8 ± 2.8 Ca	77.8 ± 4.7 Ba	94.4 ± 2.4 Aa	100.0 ± 0.0 Aa	100.0 ± 0.0 Aa	100.0 ± 0.0 Aa	100.0 ± 0.0 Aa	115.5	<0.01
*Elwendia persica*	1.1 ± 1.1 Cc	4.4 ± 2.9 Cc	7.8 ± 4.3 BCc	14.4 ± 4.4 ABc	21.1 ± 3.9 Ac	21.1 ± 3.9 Ac	26.7 ± 4.4 A c	12.3	<0.01
pirimiphos-methyl	10.0 ± 1.7 Db	13.3 ± 2.4 CDb	24.4 ± 3.8 BCb	35.6 ± 2.4 ABb	44.4 ± 2.4 ABb	56.7 ± 3.3 Ab	68.9 ± 1.1 Ab	19.8	<0.01
*F*	29.6	34.3	51.7	75.7	386.9	440.7	159.3		
*p*	<0.01	<0.01	<0.01	<0.01	<0.01	<0.01	<0.01		

For each EO, within each row, means followed by the same uppercase letter are not significantly different (df = 6, 62; Tukey HSD test at *p* = 0.05). For each concentration, within each column, means followed by the same lowercase letter are not significantly different (df = 4, 44; Tukey HSD test at *p* = 0.05). No significant differences were recorded where no letters exist. Due to zero values, mortality data for larvae exposed to *C. maritimum*, *T. ammi*, and *E. persica* EOs at 500 ppm are not shown. Due to zero values, mortality data for larvae exposed to *T. ammi* EO at 1000 ppm are not shown. Due to zero values, mortality data for adults exposed to *C. maritimum* EO at 500 ppm are not shown.

**Table 7:** Mean (%) mortality ± standard errors (SE) of *Trogoderma granarium* larvae and adults after 1–7 days in wheat treated with *Crithmum maritimum*, *Smyrnium olusatrum*, *Trachyspermum ammi*, and *Elwendia persica* essential oils (EOs) at two concentrations, with positive control, pirimiphos–methyl.

Exposure	1 Day	2 Days	3 Days	4 Days	5 Days	6 Days	7 Days		
				Larvae					
EO Type	Concentration:	500 ppm						*F*	*p*
*Smyrnium olusatrum*	3.3 ± 1.7 D	7.8 ± 2.2 CDa	17.8 ± 3.6 BCa	27.8 ± 5.2 ABa	31.1 ± 5.4 ABa	47.8 ± 5.5 ABa	50.0 ± 5.0 Aa	16.3	<0.01
*Trachyspermum ammi*	3.3 ± 1.7 C	6.6 ± 1.7 BCa	17.8 ± 3.2 ABa	26.7 ± 5.5 Aa	35.6 ± 5.3 Aa	41.1 ± 5.1 Aa	41.1 ± 5.1 Aa	13.0	<0.01
pirimiphos-methyl	2.2 ± 1.5 C	3.3 ± 1.7 Cab	6.7 ± 1.7 BCa	15.6 ± 2.9 ABb	20.0 ± 2.9 Aa	23.3 ± 2.4 Ab	26.7 ± 2.9 Ab	14.8	<0.01
*F*	1.8	6.7	21.1	59.3	170.5	457.0	457.7		
*p*	0.14	0.01	<0.01	<0.01	<0.01	<0.01	<0.01		
	Concentration:	1000 ppm							
*Crithmum maritimum*	0.0 ± 0.0 Cb	0.0 ± 0.0 Cb	0.0 ± 0.0 Cb	3.3 ± 1.7 BCc	12.2 ± 4.7 ABc	14.4 ± 4.1 ABb	17.8 ± 4.0 Ac	11.3	<0.01
*Smyrnium olusatrum*	13.3 ± 3.3 Ca	32.2 ± 4.3 Ba	54.4 ± 5.3 ABa	63.3 ± 5.5 ABa	72.2 ± 4.9 Aa	86.7 ± 4.1 Aa	93.3 ± 2.9 Aa	18.9	<0.01
*Trachyspermum ammi*	8.9 ± 2.0 Ca	23.3 ± 2.9 Ba	41.1 ± 4.2 ABa	47.8 ± 4.7 ABa	55.6 ± 5.0 Aa	63.3 ± 5.3 Aa	68.9 ± 4.6 Aab	22.4	<0.01
*Elwendia persica*	0.0 ± 0.0 Bb	0.0 ± 0.0 Bb	0.0 ± 0.0 Bb	0.0 ± 0.0 Bc	0.0 ± 0.0 Bd	2.2 ± 1.5 ABc	4.4 ± 2.4 Ad	2.8	0.02
pirimiphos-methyl	1.1 ± 1.1 Cb	3.3 ± 1.7 Cb	8.9 ± 3.1 BCb	13.3 ± 2.4 ABb	17.8 ± 2.2 Ab	21.1 ± 3.9 Ab	25.6 ± 3.4 Abc	14.5	<0.01
*F*	15.0	70.1	57.4	57.7	46.9	34.6	30.7		
*p*	<0.01	<0.01	<0.01	<0.01	<0.01	<0.01	<0.01		
				**Adults**					
	Concentration:	500 ppm							
*Crithmum maritimum*	0.0 ± 0.0 Eb	5.6 ± 1.8 Dc	8.9 ± 1.1 CDc	14.4 ± 3.4 BCDc	21.1 ± 5.9 ABCc	42.2 ± 7.2 ABb	48.9 ± 6.8 Ab	21.7	<0.01
*Smyrnium olusatrum*	0.0 ± 0.0 Cb	27.8 ± 2.8 Ba	42.2 ± 5.2 Bab	70.0 ± 8.3 Aa	80.0 ± 6.7 Aa	81.1 ± 6.1 Aa	81.1 ± 6.1 Aa	274.2	<0.01
*Trachyspermum ammi*	18.9 ± 2.6 Ca	40.0 ± 4.1 Ba	57.8 ± 4.3 ABa	65.6 ± 4.4 ABa	71.1 ± 4.8 ABab	77.8 ± 4.3 Aa	78.9 ± 4.6 Aa	13.7	<0.01
*Elwendia persica*	0.0 ± 0.0 Cb	8.9 ± 2.6 Bbc	30.0 ± 3.7 Ab	32.2 ± 4.3 Ab	35.6 ± 3.4 Ab	38.9 ± 3.1 Ab	42.2 ± 2.8 Ab	51.0	<0.01
pirimiphos-methyl	13.3 ± 1.7 Da	18.9 ± 2.6 Dab	32.2 ± 2.8 Cab	43.3 ± 4.4 BCab	52.2 ± 4.0 ABab	63.3 ± 3.3 ABa	72.2 ± 3.6 Aa	45.1	<0.01
*F*	82.1	11.8	20.8	15.0	12.4	11.8	12.8		
*p*	<0.01	<0.01	<0.01	<0.01	<0.01	<0.01	<0.01		
	Concentration:	1000 ppm							
*Crithmum maritimum*	3.3 ± 1.7 Dbc	16.7 ± 3.3 Cc	33.3 ± 4.4 BCc	43.3 ± 4.7 ABb	57.8 ± 3.2 ABb	81.1 ± 3.5 ABb	91.1 ± 2.6 Aa	29.5	<0.01
*Smyrnium olusatrum*	25.6 ± 5.8 Ba	58.9 ± 5.9 Aa	91.1 ± 4.6 Aa	100.0 ± 0.0 Aa	100.0 ± 0.0 Aa	100.0 ± 0.0 Aa	100.0 ± 0.0 Aa	15.9	<0.01
*Trachyspermum ammi*	24.4 ± 5.6 Ca	44.4 ± 6.0 Bab	66.7 ± 7.5 ABab	88.9 ± 7.5 ABa	97.8 ± 2.2 Aa	98.7 ± 1.1 Aab	100.0 ± 0.0 Aa	13.6	<0.01
*Elwendia persica*	0.0 ± 0.0 Ec	16.7 ± 2.4 Dc	42.2 ± 3.2 Cbc	56.7 ± 4.7 BCb	80.0 ± 7.3 ABa	87.8 ± 6.0 Aab	87.8 ± 6.0 Aa	321.7	<0.01
pirimiphos-methyl	13.3 ± 4.7 Cab	18.9 ± 5.1 BCbc	31.1 ± 3.5 ABc	42.2 ± 4.3 ABb	54.4 ± 4.4 Ab	64.4 ± 4.8 Ac	70.0 ± 5.8 Ab	14.5	<0.01
*F*	11.0	7.6	15.7	24.2	20.3	12.4	8.6		
*p*	<0.01	0.01	<0.01	<0.01	<0.01	<0.01	<0.01		

For each EO, within each row, means followed by the same uppercase letter are not significantly different (df = 6, 62; Tukey HSD test at *p* = 0.05). For each concentration, within each column, means followed by the same lowercase letter are not significantly different (df = 4, 44; Tukey HSD test at *p* = 0.05). No significant differences were recorded where no letters exist. Due to zero values, mortality data for larvae exposed to *C. maritimum* and *E. persica* EOs at 500 ppm are not shown.

**Table 8:** Mean (%) mortality ± standard errors (SE) of *Oryzaephilus surinamensis* larvae and adults after 1–7 days in wheat treated with *Crithmum maritimum*, *Smyrnium olusatrum*, *Trachyspermum ammi*, and *Elwendia persica* essential oils (EOs) at two concentrations, with positive control, pirimiphos–methyl.

Exposure	1 Day	2 Days	3 Days	4 Days	5 Days	6 Days	7 Days		
				Larvae					
EO Type	Concentration:	500 ppm						*F*	*p*
*Crithmum maritimum*	0.0 ± 0.0 Cb	0.0 ± 0.0 Cc	3.3 ± 1.7 Cc	10.0 ± 2.4 Bb	13.3 ± 2.4 ABb	20.0 ± 2.9 ABb	26.7 ± 4.1 Aa	27.4	<0.01
*Smyrnium olusatrum*	23.3 ± 3.3 Ba	40.0 ± 5.3 ABa	47.8 ± 5.2 Aa	53.3 ± 5.0 Aa	57.8 ± 6.2 Aa	57.8 ± 6.2 Aa	57.8 ± 6.2 Aa	7.4	<0.01
*Trachyspermum ammi*	3.3 ± 1.7 Bb	10.0 ± 2.9 Bb	28.9 ± 4.2 Aab	37.8 ± 6.0 Aa	44.4 ± 4.4 Aa	47.8 ± 4.7 Aab	53.3 ± 4.1 Aa	21.0	<0.01
*Elwendia persica*	0.0 ± 0.0 Bb	0.0 ± 0.0 Bc	0.0 ± 0.0 Bc	0.0 ± 0.0 Bc	3.3 ± 1.7 ABc	5.6 ± 2.4 ABc	6.7 ± 2.4 Ab	4.7	<0.01
pirimiphos-methyl	2.2 ± 1.5 Db	10.0 ± 2.4 Cb	16.7 ± 2.4 BCb	25.6 ± 2.9 ABa	35.6 ± 4.4 ABa	45.6 ± 5.3 Aab	62.2 ± 5.2 Aa	31.2	<0.01
*F*	26.9	29.4	61.9	58.0	29.2	26.2	25.3		
*p*	<0.01	<0.01	<0.01	<0.01	<0.01	<0.01	<0.01		
	Concentration:	1000 ppm							
*Crithmum maritimum*	6.7 ± 1.7 Cb	12.2 ± 1.5 Bbc	15.6 ± 2.4 Bb	28.9 ± 4.2 ABb	38.9 ± 5.6 Ab	46.7 ± 5.8 Ab	61.1 ± 7.2 Ab	19.0	<0.01
*Smyrnium olusatrum*	34.4 ± 3.8 Ca	72.2 ± 2.8 Ba	85.6 ± 3.4 ABa	96.7 ± 1.7 Aa	100.0 ± 0.0 Aa	100.0 ± 0.0 Aa	100.0 ± 0.0 Aa	71.3	<0.01
*Trachyspermum ammi*	34.4 ± 2.9 Da	47.8 ± 2.8 Ca	67.8 ± 2.8 Ba	87.8 ± 3.2 Aa	96.7 ± 1.7 Aa	100.0 ± 10.0 Aa	100.0 ± 0.0 Aa	90.2	<0.01
*Elwendia persica*	11.1 ± 2.0 Db	14.4 ± 1.8 CDb	16.7 ± 1.7 CDb	26.7 ± 2.9 BCb	36.7 ± 3.3 ABb	46.7 ± 3.7 ABb	53.3 ± 2.9 Ab	20.1	<0.01
pirimiphos-methyl	2.2 ± 1.5 Db	8.9 ± 2.0 Cc	14.4 ± 2.4 BCb	26.7 ± 2.9 ABb	30.0 ± 2.4 ABb	43.3 ± 3.3 Ab	63.3 ± 3.7 Ab	27.5	<0.01
*F*	21.1	29.0	29.9	50.4	43.9	32.3	21.2		
*p*	<0.01	<0.01	<0.01	<0.01	<0.01	<0.01	<0.01		
				**Adults**					
	Concentration:	500 ppm							
*Crithmum maritimum*	3.3 ± 1.7 Dab	10.0 ± 1.7 Ca	17.8 ± 2.8 BCa	23.3 ± 2.4 ABCa	25.6 ± 2.4 ABCa	35.6 ± 4.1 ABa	43.3 ± 4.4 Aa	16.7	<0.01
*Smyrnium olusatrum*	14.4 ± 4.4 Ba	21.1 ± 4.8 ABa	27.8 ± 5.2 Aa	30.0 ± 5.0 Aa	33.3 ± 4.4 Aa	35.6 ± 4.4 Aa	41.1 ± 3.1 Aa	4.7	<0.01
*Trachyspermum ammi*	0.0 ± 0.0 Db	12.2 ± 1.5 Ca	27.8 ± 2.8 Ba	34.4 ± 3.4 ABa	37.8 ± 3.6 ABa	42.2 ± 4.0 Aa	48.9 ± 3.5 Aa	232.0	<0.01
*Elwendia persica*	5.6 ± 1.8 Cab	8.9 ± 2.6 BCab	14.4 ± 2.9 ABCa	18.9 ± 2.6 ABa	25.6 ± 2.9 Aa	30.0 ± 33.3 Aa	33.3 ± 1.7 Aa	9.3	<0.01
pirimiphos-methyl	2.2 ± 1.5 Bab	2.2 ± 1.5 Bb	3.3 ± 1.7 ABb	6.7 ± 1.7 ABb	7.8 ± 1.5 ABb	8.9 ± 1.1 Ab	8.9 ± 1.1 Ab	4.5	<0.01
*F*	4.1	7.0	12.1	12.6	13.6	15.0	26.9		
*p*	0.01	0.01	<0.01	<0.01	<0.01	<0.01	<0.01		
	Concentration:	1000 ppm							
*Crithmum maritimum*	12.2 ± 2.2 Da	25.6 ± 3.8 Ca	36.7 ± 4.7 BCb	53.3 ± 4.4 ABa	56.7 ± 4.7 ABa	72.2 ± 4.0 Aa	77.8 ± 4.3 Aa	20.7	<0.01
*Smyrnium olusatrum*	26.7 ± 4.7 Ca	36.7 ± 5.8 BCa	48.9 ± 5.9 ABCb	60.0 ± 5.0 ABa	67.8 ± 4.7 ABa	71.1 ± 4.6 ABa	75.6 ± 3.4 Aa	5.0	<0.01
*Trachyspermum ammi*	0.0 ± 0.0 Db	26.7 ± 4.1 Ca	52.2 ± 4.3 Bb	64.4 ± 4.8 ABa	67.8 ± 4.3 ABa	68.9 ± 3.5 ABa	76.7 ± 2.4 Aa	315.9	<0.01
*Elwendia persica*	10.0 ± 1.7 Da	22.2 ± 2.8 Ca	31.1 ± 2.6 BCb	42.2 ± 4.0 ABa	48.9 ± 3.1 ABa	63.3 ± 3.7 Aa	65.6 ± 3.8 Aa	26.5	<0.01
pirimiphos-methyl	1.1 ± 1.1 b	2.2 ± 1.5 b	3.3 ± 1.7 a	6.7 ± 3.7 b	7.8 ± 3.6 b	8.9 ± 3.9 b	11.1 ± 3.5 b	1.4	0.21
*F*	22.6	18.7	39.0	32.1	29.2	28.6	25.1		
*p*	<0.01	<0.01	<0.01	<0.01	<0.01	<0.01	<0.01		

For each EO, within each row, means followed by the same uppercase letter are not significantly different (df = 6, 62; Tukey HSD test at *p* = 0.05). For each concentration, within each column, means followed by the same lowercase letter are not significantly different (df = 4, 44; Tukey HSD test at *p* = 0.05). No significant differences were recorded where no letters exist.

**Table 9:** Mean (%) mortality ± standard errors (SE) of *Rhyzopertha dominica* adults after 1–7 days in wheat treated with *Crithmum maritimum*, *Smyrnium olusatrum*, *Trachyspermum ammi*, and *Elwendia persica* essential oils (EOs) at two concentrations, with positive control, pirimiphos–methyl.

Exposure	1 Day	2 Days	3 Days	4 Days	5 Days	6 Days	7 Days		
EO Type	Concentration	500 ppm						*F*	*p*
*Crithmum maritimum*	2.2 ± 1.5 Bb	8.9 ± 2.6 ABc	11.1 ± 2.0 Ab	15.6 ± 2.9 Ab	15.6 ± 2.9 Ab	16.7 ± 3.3 Ac	17.8 ± 3.2 Ac	4.6	<0.01
*Smyrnium olusatrum*	5.6 ± 2.4 Ca	13.3 ± 4.1 BCb	26.7 ± 4.4 ABab	31.1 ± 4.6 ABab	33.3 ± 4.7 ABab	36.7 ± 4.4 ABab	41.1 ± 4.6 Ab	6.1	<0.01
*Elwendia persica*	3.3 ± 1.7 Cb	6.7 ± 1.7 BCc	12.2 ± 2.2 ABab	17.8 ± 1.5 Aab	23.3 ± 2.4 Aab	24.4 ± 1.8 Abc	24.4 ± 1.8 Abc	13.9	<0.01
pirimiphos-methyl	7.8 ± 2.2 Da	20.0 ± 2.4 Ca	30.0 ± 2.9 BCa	44.4 ± 1.8 ABa	54.4 ± 2.9 ABa	68.9 ± 3.1 Aa	80.0 ± 2.9 Aa	29.5	<0.01
*F*	3.0	9.2	25.2	37.1	40.0	85.7	98.3		
*p*	0.03	<0.01	<0.01	<0.01	<0.01	<0.01	<0.01		
	Concentration:	1000 ppm							
*Crithmum maritimum*	18.9 ± 2.0 Ca	42.2 ± 2.8 Bab	50.0 ± 2.9 ABb	53.3 ± 3.7 ABb	61.1 ± 4.8 Ab	65.6 ± 5.6 Ab	68.9 ± 4.6 Abc	29.4	<0.01
*Smyrnium olusatrum*	24.4 ± 1.8 Ca	57.8 ± 3.2 Ba	83.3 ± 3.7 Aa	88.9 ± 3.1 Aa	92.2 ± 2.2 Aa	95.6 ± 1.8 Aa	95.6 ± 1.8 Aa	133.4	<0.01
*Trachyspermum ammi*	17.8 ± 2.8 Da	22.2 ± 2.8 CDbc	26.7 ± 3.3 BCDcd	32.2 ± 4.3 ABCc	35.6 ± 4.1 ABCc	41.1 ± 3.1 ABc	45.6 ± 2.4 Ad	8.0	<0.01
*Elwendia persica*	6.7 ± 1.7 Db	11.1 ± 2.6 CDd	18.9 ± 2.6 BCd	31.1 ± 4.6 ABc	37.8 ± 4.9 ABc	43.3 ± 6.2 ABc	56.7 ± 6.5 Acd	13.5	<0.01
pirimiphos-methyl	4.4 ± 2.4 Db	17.8 ± 3.6 Ccd	30.0 ± 2.9 BCc	41.1 ± 2.6 ABbc	53.3 ± 2.4 ABb	68.9 ± 2.6 Aab	82.2 ± 4.0 Aab	41.6	<0.01
*F*	13.0	13.8	29.1	17.0	21.0	18.6	21.0		
*p*	<0.01	<0.01	<0.01	<0.01	<0.01	<0.01	<0.01		

For each EO, within each row, means followed by the same uppercase letter are not significantly different (df = 6, 62; Tukey HSD test at *p* = 0.05). For each concentration, within each column, means followed by the same lowercase letter are not significantly different (df = 4, 44; Tukey HSD test at *p* = 0.05). Due to zero values, mortality data for adults exposed to *T. ammi* EO at 500 ppm are not shown.

**Table 10:** Mean (%) mortality ± standard errors (SE) of *Sitophilus oryzae* adults after 1–7 days in wheat treated with *Crithmum maritimum*, *Smyrnium olusatrum*, *Trachyspermum ammi*, and *Elwendia persica* essential oils (EOs) at two concentrations, with positive control, pirimiphos–methyl.

Exposure	1 Day	2 Days	3 Days	4 Days	5 Days	6 Days	7 Days		
EO Type	Concentration:	500 ppm						*F*	*p*
*Crithmum maritimum*	3.3 ± 1.7 Cbc	10.0 ± 1.7 Bc	14.4 ± 1.8 ABc	17.8 ± 2.2 ABCc	28.9 ± 3.5 Ab	31.1 ± 3.1 Ab	33.3 ± 3.3 Ab	21.3	<0.01
*Smyrnium olusatrum*	20.0 ± 3.3 Ba	48.9 ± 3.9 Aa	56.7 ± 2.4 Aa	68.9 ± 2.0 Aa	85.6 ± 4.1 Aa	87.8 ± 3.6 Aa	90.0 ± 2.9 Aa	18.3	<0.01
*Elwendia persica*	0.0 ± 0.0 Bc	0.0 ± 0.0 Bd	0.0 ± 0.0 Bd	0.0 ± 0.0 Bd	3.3 ± 1.7 ABc	6.7 ± 2.4 Ac	6.7 ± 2.4 Ac	5.6	<0.01
pirimiphos-methyl	10.0 ± 2.9 Dab	22.2 ± 2.2 Cb	40.0 ± 2.4 BCb	47.8 ± 2.8 ABb	65.6 ± 2.9 ABa	74.4 ± 3.8 ABa	91.1 ± 3.1 Aa	31.5	<0.01
*F*	17.1	171.1	1078.4	1081.3	117.2	88.9	94.1		
*p*	<0.01	<0.01	<0.01	<0.01	<0.01	<0.01	<0.01		
	Concentration:	1000 ppm							
*Crithmum maritimum*	17.8 ± 3.6 Da	38.9 ± 5.1 Cab	52.2 ± 6.2 BCab	67.8 ± 7.4 ABa	78.9 ± 6.3 ABa	86.7 ± 6.7 Aa	94.4 ± 4.4 Aa	30.9	<0.01
*Smyrnium olusatrum*	82.2 ± 5.5 Ba	97.8 ± 1.5 Aa	100.0 ± 0.0 Aa	100.0 ± 0.0 Aa	100.0 ± 0.0 Aa	100.0 ± 0.0 Aa	100.0 ± 0.0 Aa	12.7	<0.01
*Trachyspermum ammi*	3.3 ± 1.7 bc	3.3 ± 1.7 c	4.4 ± 1.8 c	5.6 ± 2.4 b	6.7 ± 2.4 b	7.8 ± 2.2 b	8.9 ± 2.0 b	1.1	0.39
*Elwendia persica*	0.0 ± 0.0 Bc	0.0 ± 0.0 Bd	0.0 ± 0.0 Bd	3.3 ± 1.7 ABb	3.3 ± 1.7 ABb	6.7 ± 1.7 Ab	11.1 ± 3.1 Ab	6.1	<0.01
pirimiphos-methyl	8.9 ± 2.0 Dab	24.4 ± 2.4 Cb	38.9 ± 2.0 BCb	50.0 ± 2.4 ABa	65.6 ± 3.4 ABa	77.8 ± 4.3 Aa	93.3 ± 4.1 Aa	35.2	<0.01
*F*	12.6	96.6	103.8	43.3	44.5	34.8	28.4		
*p*	<0.01	<0.01	<0.01	<0.01	<0.01	<0.01	<0.01		

For each EO, within each row, means followed by the same uppercase letter are not significantly different (df = 6, 62; Tukey HSD test at *p* = 0.05). For each concentration, within each column, means followed by the same lowercase letter are not significantly different (df = 4, 44; Tukey HSD test at *p* = 0.05). No significant differences were recorded where no letters exist. Due to zero values, mortality data for adults exposed to *T. ammi* EO at 500 ppm are not shown.

**Table 11:** Mean (%) mortality ± standard errors (SE) of *Acarus siro* nymphs and adults after 1–7 days in wheat treated with *Crithmum maritimum*, *Smyrnium olusatrum*, *Trachyspermum ammi*, and *Elwendia persica* essential oils (EOs) at two concentrations, with positive control, pirimiphos–methyl.

Exposure	1 Day	2 Days	3 Days	4 Days	5 Days	6 Days	7 Days		
				Nymphs					
EO Type	Concentration:	500 ppm						*F*	*p*
*Crithmum maritimum*	0.0 ± 0.0 D	1.1 ± 1.1 CD	3.3 ± 1.7 BCDb	6.7 ± 2.9 ABCDbc	11.1 ± 3.9 ABCbc	13.3 ± 4.4 ABbc	17.8 ± 4.5 Ab	6.4	<0.01
*Smyrnium olusatrum*	1.1 ± 1.1 C	1.1 ± 1.1 C	3.3 ± 1.7 Cb	17.8 ± 2.8 Ba	24.4 ± 2.9 ABa	40.0 ± 7.1 ABa	55.6 ± 6.3 Aa	48.2	<0.01
*Trachyspermum ammi*	1.1 ± 1.1 C	1.1 ± 1.1 C	2.2 ± 2.2 Cb	14.4 ± 3.4 Bab	18.9 ± 3.5 ABab	30.0 ± 5.5 ABab	47.8 ± 4.0 Aa	37.6	<0.01
*Elwendia persica*	0.0 ± 0.0 C	0.0 ± 0.0 C	0.0 ± 0.0 Cb	1.1 ± 1.1 BCc	3.3 ± 1.7 ABCc	5.6 ± 1.8 ABc	6.7 ± 1.7 Ab	5.7	<0.01
pirimiphos-methyl	1.1 ± 1.1 C	3.3 ± 1.7 C	11.1 ± 2.0 Ba	21.1 ± 3.9 ABa	27.8 ± 5.2 ABa	42.2 ± 5.2 Aa	52.2 ± 4.7 Aa	35.4	<0.01
*F*	0.5	1.1	7.2	14.5	11.7	11.9	17.2		
*p*	0.74	0.35	0.01	<0.01	<0.01	<0.01	<0.01		
	Concentration:	1000 ppm							
*Crithmum maritimum*	2.2 ± 1.5 C	6.7 ± 2.9 BCab	10.0 ± 3.3 ABCa	16.7 ± 5.3 ABCa	22.2 ± 5.7 ABab	25.6 ± 5.6 ABab	32.2 ± 7.4 Aab	5.0	<0.01
*Smyrnium olusatrum*	3.3 ± 1.7 C	7.8 ± 1.5 Ba	11.1 ± 2.0 Ba	35.6 ± 4.4 Aa	43.3 ± 5.3 Aa	52.2 ± 4.9 Aa	63.3 ± 4.1 Aa	26.3	<0.01
*Trachyspermum ammi*	1.1 ± 1.1 C	5.6 ± 1.8 Cab	7.8 ± 2.2 BCa	28.9 ± 6.3 ABa	34.4 ± 7.1 Aa	44.4 ± 5.0 Aa	56.7 ± 6.7 Aa	16.8	<0.01
*Elwendia persica*	0.0 ± 0.0 B	0.0 ± 0.0 Bb	0.0 ± 0.0 Bb	3.3 ± 2.4 Bb	6.7 ± 2.4 ABb	12.2 ± 3.2 Ab	15.6 ± 4.4 Ab	9.6	<0.01
pirimiphos-methyl	1.1 ± 1.1 D	2.2 ± 1.5 Dab	7.8 ± 1.5 Ca	15.6 ± 2.4 BCa	25.6 ± 4.1 ABCa	41.1 ± 3.1 ABa	53.3 ± 2.9 Aa	25.3	<0.01
*F*	1.1	1.0	6.4	9.1	5.4	7.8	7.9		
*p*	0.38	0.01	0.01	<0.01	0.01	<0.01	<0.01		
				**Adults**					
	Concentration:	500 ppm							
*Crithmum maritimum*	2.2 ± 1.5 D	4.4 ± 1.8 CDb	7.8 ± 2.2 BCDb	12.2 ± 2.8 ABCbc	17.8 ± 3.6 ABbc	21.1 ± 4.2 ABbc	26.7 ± 4.7 Ab	8.0	<0.01
*Smyrnium olusatrum*	2.2 ± 1.5 C	3.3 ± 1.7 Cb	7.8 ± 2.2 Cb	22.2 ± 4.9 Bab	30.0 ± 5.8 ABab	46.7 ± 7.3 ABa	67.8 ± 2.8 Aa	26.7	<0.01
*Trachyspermum ammi*	2.2 ± 1.5 B	4.4 ± 5.3 Bb	7.8 ± 2.8 Bb	22.2 ± 3.2 Aab	26.7 ± 4.1 Aab	37.8 ± 4.7 Aab	55.6 ± 4.8 Aa	22.1	<0.01
*Elwendia persica*	0.0 ± 0.0 D	0.0 ± 0.0 Db	2.2 ± 1.5 CDb	5.6 ± 2.4 BCDc	8.9 ± 3.1 ABCc	13.3 ± 2.9 ABc	15.6 ± 2.4 Ac	12.0	<0.01
pirimiphos-methyl	4.4 ± 1.8 D	15.6 ± 1.8 Ca	27.8 ± 2.8 BCa	43.3 ± 3.3 ABa	58.9 ± 4.6 ABa	65.6 ± 3.4 Aa	72.2 ± 2.8 Aa	40.1	<0.01
*F*	1.3	9.6	7.1	11.3	10.2	13.3	37.7		
*p*	0.29	<0.01	0.01	<0.01	<0.01	<0.01	<0.01		
	Concentration:	1000 ppm							
*Crithmum maritimum*	3.3 ± 1.7 C	8.9 ± 3.5 BCab	15.6 ± 3.8 ABab	24.4 ± 5.6 Aab	31.1 ± 7.5 Aab	36.7 ± 8.8 Aab	45.6 ± 9.6 Aab	11.3	<0.01
*Smyrnium olusatrum*	4.4 ± 2.4 D	8.9 ± 3.1 CDab	15.6 ± 4.4 BCab	30.0 ± 6.0 ABa	38.9 ± 4.2 ABa	55.6 ± 5.8 Aa	76.7 ± 5.3 Aa	16.4	<0.01
*Trachyspermum ammi*	3.3 ± 2.4 C	7.8 ± 2.8 BCab	12.2 ± 4.0 BCab	28.9 ± 6.1 ABa	36.7 ± 7.6 ABa	52.2 ± 7.8 Aa	65.6 ± 10.4 Aa	8.3	<0.01
*Elwendia persica*	0.0 ± 0.0 C	0.0 ± 0.0 Cb	4.4 ± 1.8 BCb	10.0 ± 4.1 ABCb	14.4 ± 5.3 ABb	20.0 ± 4.7 ABb	23.3 ± 5.5 Ab	9.2	<0.01
pirimiphos-methyl	5.6 ± 1.8 D	16.7 ± 2.4 Ca	27.8 ± 4.0 BCa	42.2 ± 4.9 ABa	53.3 ± 4.7 ABa	65.6 ± 4.4 Aa	73.3 ± 3.7 Aa	31.2	<0.01
*F*	1.7	6.7	4.2	5.0	4.4	6.8	8.1		
*p*	0.17	0.01	0.01	0.01	0.01	0.01	<0.01		

For each EO, within each row, means followed by the same uppercase letter are not significantly different (df = 6, 62; Tukey HSD test at *p* = 0.05). For each concentration, within each column, means followed by the same lowercase letter are not significantly different (df = 4, 44; Tukey HSD test at *p* = 0.05). No significant differences were recorded where no letters exist.

## Data Availability

Data are available within the article.
